# Methodological quality and reporting standards in systematic reviews with meta-analysis of physical activity studies: a report from the Strengthening the Evidence in Exercise Sciences Initiative (SEES Initiative)

**DOI:** 10.1186/s13643-021-01845-9

**Published:** 2021-12-02

**Authors:** Nórton Luís Oliveira, Cíntia Ehlers Botton, Angélica Trevisan De Nardi, Daniel Umpierre

**Affiliations:** 1 National Institute of Science and Technology for Health Technology Assessment (IATS/HCPA), Hospital de Clínicas de Porto Alegre, Clinical Research Center, Porto Alegre, RS Brazil; 2 Exercise Pathophysiology Research Laboratory, Hospital de Clínicas de Porto Alegre, Clinical Research Center, Rua Ramiro Barcelos, 2350, Porto Alegre, RS CEP: 90035-903 Brazil; 3 Graduate Program in Cardiology and Cardiovascular Sciences, Universidade Federal do Rio Grande do Sul, Porto Alegre, RS Brazil; 4 Department of Public Health, Universidade Federal do Rio Grande do Sul, Porto Alegre, RS Brazil

**Keywords:** Systematic reviews with meta-analysis, Methodological quality, Reporting standards, Exercise sciences

## Abstract

**Background:**

Several resources have been developed (e.g., reporting guidelines) to promote high-standard practices in health research. However, there was no continuous and systematic assessment of recommended practices in published systematic reviews with meta-analysis (SRMAs), which increases the usability of the available resources. Therefore, we aimed to assess the methodological and reporting standards in SRMAs of physical activity studies. This report presents the main results of the SEES Initiative in 2019.

**Methods:**

Our approach is based on a prospective systematic review methodology to implement post-publication surveillance of research practices in exercise sciences. Briefly, during the year 2019, pre-specified searches were conducted monthly (PubMed/MEDLINE) in journals from the exercise sciences (*n* = 9) and medicine (*n* = 5). The assessments were independently conducted by two authors, based on 36 items/practices derived from established statements/tools (PRISMA, AMSTAR 2, ROBIS). To be eligible, SRMAs should summarize studies that had, at least, one arm consisting of physical activity interventions/exposures and one health or behavioral outcome.

**Results:**

Out of 1028 studies assessed for eligibility, 103 SRMAs were included. The minimum adherence was 13/36 items, whereas only one SRMA adhered to all items. Some highly contemplated items included identification of title as SRMA (97.1%) and descriptions of the main outcome in the abstract (95.1%) and risk of bias (RoB) assessment (95.1%). Some poorly contemplated items included publicly available protocol (4.9%), discussion of the results in light of RoB in studies included (32.0%), and data sharing statements (35.9%).

**Conclusion:**

In summary, there is a suboptimal adherence to recommended practices on methodological quality and reporting standards in the SRMAs of physical activity intervention/exposure evaluated from the selected journals in 2019, which likely reduce the reproducibility and usefulness of these studies. This incipient evidence from our first 12 months of post-publication surveillance should serve as a call for attention and action for multiple stakeholders (e.g., authors, reviewers, editors, funders, academic institutions) in this important health research field.

**Supplementary Information:**

The online version contains supplementary material available at 10.1186/s13643-021-01845-9.

## Introduction

Over the last decades, there has been a consistent increase in the publication of systematic reviews with or without meta-analysis in the health sciences [1–3]. This type of literature review is accepted as a valuable source of evidence [4–6], being highly cited [7]. Additionally, systematic reviews with meta-analysis (SRMAs) are used to inform clinical decision-making, development of clinical practice guidelines and health policies [2, 8]. In the health sciences, numerous SRMAs have been poorly conducted with low methodological quality and poor reporting [2, 9–11]. In the exercise sciences, SRMAs and clinical trials are essential to inform strategies of prevention and treatment in several clinical conditions [12, 13]; however, methodological and reproducibility issues have been scarcely addressed [14–16]. Such evidence of low-quality practices affects reproducibility, since it makes findings less verifiable, credible, and informative [17], with likely waste of financial or non-financial resources [18].

Several strategies have been developed to improve the quality of evidence syntheses, such as open handbooks for standardized methods [19], registry platforms (e.g., International Prospective Register of Systematic Review (PROSPERO)), data sharing policies, reporting guidelines, and methodological quality assessment tools [20]. In this context, the “Preferred Reporting Items for Systematic Reviews and Meta-Analyses Statement” (PRISMA Statement) [21], a reporting guideline, and the “A MeaSurement Tool to Assess systematic Reviews” (AMSTAR 2) [22], a methodological quality assessment tool, are carefully conceived documents to improve the quality of SRMAs. However, reproducibility requires methodological rigor, adequate reporting, and transparency in the design, conduction, analysis, and critical appraisal of scientific evidence [18, 23], denoting that adherence to existent resources is of foremost importance. Since adherence is still quite low [1, 2], advocacy, education/training, implementation, and surveillance are issues to be addressed in a more intense and in-depth way [1, 2, 24, 25].

Thus, we have developed and implemented a discipline-based initiative with a surveillance approach to monthly evaluate the practices from the publications in exercise sciences, named SEES Initiative. Specifically, our purpose was to prospective and systematically assess methodological and reporting standards of systematic reviews with meta-analyses of physical activity from selected journals in the field of exercise sciences and general medicine, as well as to disseminate our findings publicly and provide direct feedback to authors and journal editors.

The present report brings the initial evidence of our first 12 months of assessments in 2019 in order to attract the attention of different stakeholders to the adherence or not of relevant aspects of the quality standard (methodological and reporting) in a considerable sample of SRMAs in the exercise sciences published in top-tier journals and, finally, show the importance of conducting such initiative.

## Methods

Institutional review board approval and informed consent were not required, since this study used publicly available data from the SRMAs included. The Preferred Reporting Items for Systematic Reviews and Meta-Analyses: The PRISMA Statement and the PRISMA checklist were used to ensure accurate reporting (see Additional file 1).

### Design and organization

The Strengthening the Evidence in Exercise Sciences Initiative (SEES Initiative) is a collaborative, nonprofit, living project for assessment of published research (i.e., randomized controlled trials and SRMAs) and dissemination of recommended practices of transparency, reproducibility, and integrity. This project was launched in January 2019, and the results to be presented herein refer to the SRMA assessments from January to December 2019. Our protocol is available at the Open Science Framework repository (OSF) [26].

The SEES Initiative is conducted by trained collaborators organized in three committees. The pre-assessment committee is responsible for literature searches in selected journals and retrieval. The assessment committee carries out the assessment of eligibility criteria and conducts data extraction on randomized controlled trials and SRMAs. The post-assessment committee conducts the data management (i.e., analyses, storage, and sharing) and dissemination of evidence appraisal to authors, journal editors, and websites. To ensure internal consistency, planned tasks of each committee follow standardized operational procedures (https://osf.io/efgvy/).

These collaborators do not have exclusive dedication or receive any payment. Therefore, to achieve the SEES Initiative ambitious aim of continuously conducting post-publication surveillance, disseminating the findings publicly, and providing direct feedback to authors and journal editors, we had to select and limit the number of journals to carry out literature searches. The criteria used for the journal’s selection are described in the section “Literature search”.

Our methodological design relates to a meta-research prospective approach, mostly regarding post-publication analyses and, therefore, submission to ethical committees is not applicable for this study design.

### Eligibility criteria

We included SRMAs which synthesized primary studies having at least one intervention arm of physical activity or exercise programs with a research question related to health outcomes or health behaviors at a minimal extent. In addition, SRMAs of observational studies with well-defined physical activity exposures were eligible for inclusion, as well as reviews with exploratory approaches (e.g., meta-regression) using meta-analytic techniques.

### Literature search

We conducted searches in PubMed/MEDLINE between the 3rd and 7th day of each month of 2019. To reduce the burden with eligibility analyses, after conducting pilot tests and examining specific literature, search strategies (see Additional file 2) were built using highly sensitive filters for SRMAs [27, 28]. Moreover, we applied date filters restricting the searches for the two past months. For example, February was included in the search conducted both in March as well as in April. As expected, this strategy resulted in duplicated references because each month was looked up twice. Since MEDLINE/PubMed does have some timing variability in indexing of references, we implemented such overlapping procedure because non-overlapped searches yielded loss of some references in our pilot tests.

The searches were conducted and retrieved on a monthly basis in 9 exercise science journals (*American Journal of Sports Medicine*, *British Journal of Sports Medicine*, *European Journal of Preventive Cardiology*, *International Journal of Behavioral Nutrition and Physical Activity*, *Journal of Physiotherapy*, *Journal of Science and Medicine in Sport*, *Medicine and Science in Sports and Exercise*, *Scandinavian Journal of Medicine & Science in Sports and Sports Medicine*) and on a quarterly basis from 5 general medicine journals (*Annals of Internal Medicine*, *British Medical Journal*, *Journal of the American Medical Association*, *Lancet*, and *New England Journal of Medicine*). The choice of these exercise science journals was guided by the audience reach, for which we considered whether a given journal was linked to a professional or scientific society, journal impact factor, and by the expected volume of SRMA publications. Regarding the general medicine journals, our choice was based on the public reach and media coverage that articles addressing physical activity exposures/interventions usually achieve whenever published by these journals. The protocol document presents further reasoning regarding the journals’ choice [26].

### Screening

Two trained collaborators (N.L.O and C.E.B) composed the assessment committee for SRMAs and screened independently the title/abstracts for eligibility. The same collaborators read the full texts of potential eligible SRMAs for a final selection. Any disagreements were solved by a third collaborator (D.U.). Full data set with publications assessed for eligibility is publicly available on OSF (https://osf.io/6jzb8/).

### Data extraction (SRMA assessment)

We set up a standardized form to assess the methodological and reporting quality of the SRMAs, which was completed independently by two collaborators (N.L.O and C.E.B). Disagreements were resolved with participation of a third collaborator (D.U.). The assessment form was constructed based on documents widely available and referred to in the literature. The questions were therefore operationalized based on recommendation items from the PRISMA Statement [21], the AMSTAR 2 [22], and the “Risk of Bias in Systematic Reviews” (ROBIS) [29]. Additional file 3 presents the items included in the assessment form and respective guideline/tools of reference.

In this report, methodological quality refers to how well SRMA was planned and conducted, taking into account the processes of analysis, synthesis, and critical appraisal of the selected evidence [22]. In addition, the quality of reporting refers to the details and completeness of the information throughout the research report [21].

### Items and domains

The SRMAs (unit of analysis) were analyzed by (1) cumulative number of items (from 0 to 36) in accordance with recommended practices and (2) seven domains that aggregate these items based on contributions in methodological or reporting aspects. The seven domains were transparency (*n* = 4 items), completeness (*n* = 11), methodological rigor (*n* = 7), participants (*n* = 2), interventions/exposures (*n* = 2), outcome (*n* = 5), and critical appraisal (*n* = 5). Although the domains are intrinsically related, items were distributed in an exclusive approach to avoid overlapped counts (which is only present for one item associated with two domains in the SRMA assessment) (see Additional file 3). We underscore that the “domains” are labeled as “components” both on the website and in the SEES Initiative protocol [26]. In this report, registration and methodological protocol were considered separately. When we cite “publicly available methodological protocol,” we are referring to a detailed protocol that allows the reproducibility of the study and is publicly available, but not necessarily published in a journal. This document may be available on an OSF page, Google Drive link, institutional webpages, Google sites, among others.

### Dissemination of individual results

After SRMA assessments were completed, we disseminated reports by three means: (1) summarized reports at our website (www.sees-initiative.org), (2) deposit of full reports were filed in OSF (https://osf.io/ntw7d/ folder: assessment reports/SRMA), and (3) full reports sent by email to the corresponding authors. We encouraged authors to send us requests for clarification or correction.

### Statistical analysis

A general summary is presented in median and interquartile range (25th–75th percentiles). Data for each item evaluated are presented as absolute frequencies (*n*) and percentages of SRMA publications that received each possible answer (for example, yes or no). Regarding the domains, the data are summarized as frequencies and percentages of SRMAs that scored as “Yes” or a “positive” evaluation in a specific number of items in each individual domain. In most items, the option “Yes” was indicative of a recommended practice (item) that was followed in the SRMA. Items that did not have a binary (yes/no) response option included those related to the time range of literature search, duplicated process for selection, extraction, and assessment of risk of biases (yes vs. partially yes), number of languages considered for study eligibility, description of risk of bias within studies, description for protocol deviations during the course of the review, and discussion limitations at the study/outcome and/or at the review level. Another type of exception regards the occurrence of a potential spin bias, in which “No” is the positive (recommended) evaluation. We describe these exceptions in detail and their possible operationalization in Additional file 4.

Although we did compute a cumulative frequency of adherence to recommended practices per study, we chose not to disseminate study results primarily based on scores, grades, or single index, because the use of such metrics can hide critical flaws in important items for reproducibility, credibility, and trustworthiness of the SRMA findings [22]. In this sense, we attempt to communicate such frequencies by also showing all the items included in the assessment.

An exploratory analysis was performed to evaluate whether the adherence to a recommended practice (item) is different between SRMAs registered and not registered a priori and between SRMAs with open access and without open access. The chi-square test was used for this analysis. However, when at least 80% of the expected frequencies (counts) are not equal to or greater than 5, the Fisher exact test was used.

The data was entered into a Google Sheet, which was generated from a standardized Google Form, filled in duplicate. All variable manipulation and related analyses were carried out using R software version 4.0.0 (R Development Core Team, Vienna, Austria). The R script and data are openly available in our study repository (https://osf.io/ntw7d/). Statistical significance was set at *p* < 0.05.

## Results

**Fig. 1:**
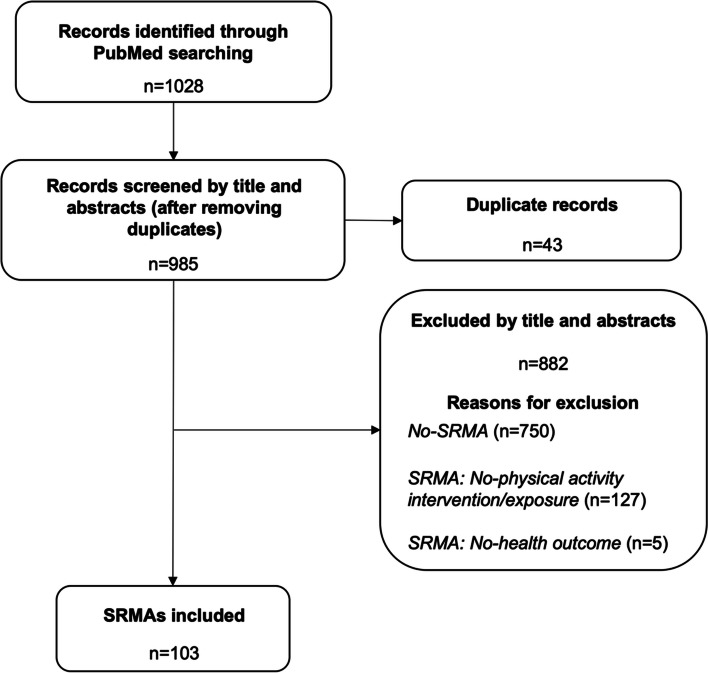
Flow diagram of the study selection process. SRMAs, systematic reviews with meta-analysis

A total of 1028 records were retrieved from the electronic literature searches performed monthly in 2019. Nine hundred and eighty-five were screened by title and abstract, and 882 were excluded. Thus, after confirmation by full-text screening, 103 SRMAs were eligible for assessment (Fig. 1). Only one SRMA included was published in general medicine journals. Most publications were from *Sports Medicine* (*n* = 36) and *British Journal of Sports Medicine* (*n* = 34) (see Additional file 5).

**Fig. 2:**
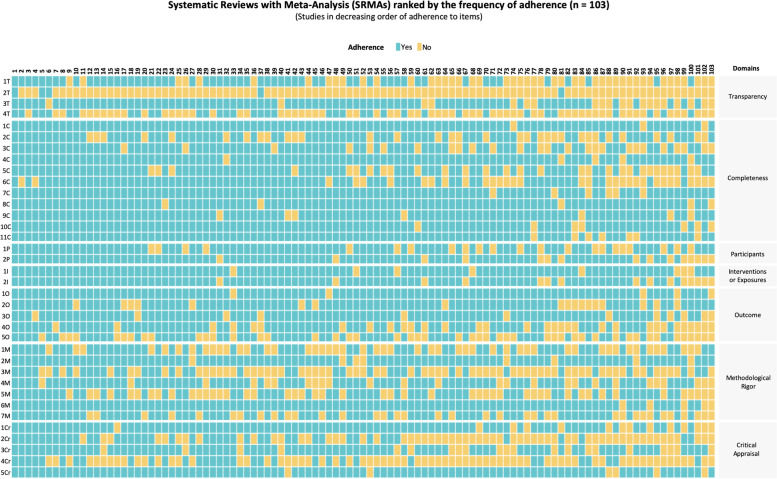
Overview of the adherence to recommended practices: items (*n* = 36) per SRMA (*n* = 103). Rows refer to items/recommended practices (*n* = 36) and columns refer to systematic reviews with meta-analysis (*n* = 103). The studies are organized in descending order of adherence to the items/recommended practices (from left to right). **Four items of transparency:** 1T: Registration; 2T: Protocol; 3T: Available searches; 4T: Data Statement. **Eleven items of completeness:** 1C: Title as SRMA; 2C: Data sources (abstract); 3C: Key eligibility criteria (abstract); 4C: Number of included studies (abstract); 5C: Research question; 6C: PICOS explanation; 7C: Number of references; 8C: Description of sample sizes; 9C: Duration of included studies; 10C: Sources of funding; 11C: Potential conflicts of interest. **Two items of participants:** 1P: Description of participants (abstract); 2P: Detailed studies' characteristics. **Two items of interventions/exposures:** 1I: Description of interventions/exposures (ab); 2I: Detailed studies' characteristics. **Five items of outcome:** 1O: Main outcome of interest (abstract); 2O: Statistical methods; 3O: Statistical heterogeneity; 4O: Meta-analytic summary estimates; 5O: Statistics per study. **Seven items of methodological rigor** 1M: Searches in grey literature; 2M: Searches from inception or with justification; 3M: Number of languages; 4M: Study selection in duplicate; 5M: Data extraction in duplicate; 6M: Description of Risk of Bias assessment; 7M: Risk of Bias assessment in duplicate. **Five items of critical appraisal:** 1Cr: Risk of Bias results within studies; 2Cr: Description of protocol deviations; 3Cr: Presence of spin bias; 4Cr: Discussion addressing Risk of Bias; 5Cr: Limitations thoroughly addressed. The list with the identification of the studies in this same order is available in the Additional file 7

Overall, one SRMA addressed appropriately the 36 core items (Fig. 2; see Additional file 6 for more details). The median of items that received “Yes” or other “positive” evaluation was 28 items (24–29 items). The item regarding the identification as a systematic review, meta-analysis, or both in the study title (completeness domain) was the one with the highest adherence (100/103, 97.1%). On the other hand, the item regarding the existence of a publicly available methodological protocol (transparency domain) was the one with the lowest adherence (5/103, 4.9%) (Table 1).

**Table 1:** Frequency distribution of items/recommended practices by domains for the total number of systematic reviews with meta-analysis (SRMAs)

	SRMAs (***n*** = 103)
**Domain: transparency**	
***Registration***	
No	46 (44.7%)
Yes	57 (55.3%)
***Protocol***	
No	98 (95.1%)
Yes	5 (4.9%)
***Available searches***	
No	20 (19.4%)
Yes	83 (80.6%)
***Data statement***	
No	66 (64.1%)
Yes	37 (35.9%)
**Domain: completeness**	
***Title as SRMA***	
No	3 (2.9%)
Yes	100 (97.1%)
***Data sources (ab)***	
No	33 (32.0%)
Yes	70 (68.0%)
***Key eligibility criteria (ab)***	
No	26 (25.2%)
Yes	77 (74.8%)
***Number of included studies (ab)***	
No	5 (4.9%)
Yes	98 (95.1%)
***Research question***	
No	29 (28.2%)
Yes	74 (71.8%)
***PICOS explanation***	
No	32 (31.1%)
Yes	71 (68.9%)
***Number of references***	
No	6 (5.8%)
Yes	97 (94.2%)
***Description of sample sizes***	
No	5 (4.9%)
Yes	98 (95.1%)
***Duration of included studies***	
Does not apply	16 (15.5%)
No	7 (6.8%)
Yes	80 (77.7%)
***Sources of funding***	
No	7 (6.8%)
Yes	96 (93.2%)
***Potential conflicts of interest***	
No	7 (6.8%)
Yes	96 (93.2%)
**Domain: participants**	
***Description of participants (ab)***	
No	22 (21.4%)
Yes	81 (78.6%)
***Detailed studies’ characteristics***	
No	15 (14.6%)
Yes	88 (85.4%)
**Domain: intervention/exposure**	
***Description of interventions/exposures (ab)***	
No	7 (6.8%)
Yes	96 (93.2%)
***Detailed studies’ characteristics***	
No	15 (14.6%)
Yes	88 (85.4%)
**Domain: outcome**	
***Main outcome of interest (ab)***	
No	5 (4.9%)
Yes	98 (95.1%)
***Statistical methods***	
No	18 (17.5%)
Yes	85 (82.5%)
***Statistical heterogeneity***	
No	12 (11.7%)
Yes	91 (88.3%)
***Meta-analytic summary estimates***	
No	31 (30.1%)
Yes	72 (69.9%)
***Statistics per study***	
No	46 (44.7%)
Yes	57 (55.3%)
**Domain: methodological rigor**	
***Searches in gray literature***	
No	49 (47.6%)
Yes	54 (52.4%)
***Searches from inception or with justification***	
No	8 (7.8%)
Yes	95 (92.2%)
***Number of languages***	
1	62 (60.2%)
2	11 (10.7%)
3	7 (6.8%)
4	1 (1.0%)
No restriction	19 (18.4%)
No statement	3 (2.9%)
***Study selection in duplicate***	
No	26 (25.2%)
Partial yes (e.g., a sample of 50% of studies were checked by two independent researchers)	3 (2.9%)
Yes	74 (71.8%)
***Data extraction in duplicate***	
No	47 (45.6%)
Partial yes (e.g., a sample of 50% of studies were checked by two independent researchers)	1 (1.0%)
Yes	55 (53.4%)
***Description of RoB assessment***	
No	5 (4.9%)
Yes	98 (95.1%)
***RoB assessment in duplicate***	
No	35 (34.0%)
Yes	68 (66.0%)
**Domain: critical appraisal**	
***RoB results within studies***	
No	14 (13.6%)
Partial yes (there are individual results without specification of specific criteria/domains)	17 (16.5%)
Yes	72 (69.9%)
***Description of protocol deviations***	
No	18 (17.5%)
Unclear	43 (41.7%)
Yes	16 (15.5%)
Does not apply	26 (25.2%)
***Presence of spin bias***	
No	82 (79.6%)
Yes	21 (20.4%)
***Discussion addressing RoB***	
No	70 (68.0%)
Yes	33 (32.0%)
***Limitations thoroughly addressed***	
No	6 (5.8%)
Yes, BOTH for study and review levels	74 (71.9%)
Yes, ONLY for the review level (limitation within or across studies not mentioned)	2 (1.9%)
Yes, ONLY for the study and/or outcome level (review processes not mentioned)	21 (20.4%)

*ab* Abstract, *PICOS* Acronym for population, intervention, comparator/control, outcome, setting, *RoB* Risk of bias

### Transparency

For the four items of this domain, only 2.9% (3/103) of SRMAs received “yes” on all (full yes) but 11.7% (12/103) received “no” on all (full no). The item with the most positive evaluation was related to the availability of at least one full-search query (83/103, 80.6% of SRMAs) (Table 1). Just over a third of the reports made a statement regarding the data sharing (37/103, 35.9%) and just over half (57/103, 55.3%) were registered in a public database.

### Completeness

Thirty SRMAs (29.1%) complied to all the 11 items of this domain. In addition, 7 items presented a high percentage of “yes” (93.2% to 97.1%) among the SRMAs. However, 31.1% (32/103) of SRMAs did not report a detailed explanation of eligibility criteria for PICOS elements (population, intervention, comparator, outcome, setting), and the abstract of 25.2% SRMAs (26/103) did not inform some of the key eligibility criteria for study selection (Table 1).

### Participants and interventions/exposures

These two domains were composed of two items, one of which (i.e., full description of PICOS elements in the Results section) is repeated in both domains. Eighty-eight (85.4%) SRMAs scored “yes” in this item. Also, 69 (67.0%) SRMAs received full yes in the participants domain and 82 (79.6%) SRMAs in the interventions/exposures domain. However, 21.4% (22/103) of SRMAs did not provide a description regarding the population (participants) or main condition(s) addressed in the abstract section (Table 1).

### Outcome

Thirty-five SRMAs (34.0%) complied to all the 5 items of this domain. The item with the most positive evaluation was related to the result description for the main outcome in the abstract (98/103, 95.1%). However, almost half of SRMAs (46/103, 44.7%) failed to report a full description of individual results for studies composing the meta-analysis (i.e., effects size, imprecision measure, and percentage weight) in the Results section. Moreover, 30.1% (31/103) of SRMAs did not describe meta-analytic summary estimates as recommended (i.e., binary outcomes as frequencies with and without the event (or as proportions such as 12/45); continuous outcomes as the mean, standard deviation, and sample size for each group) (Table 1).

### Methodological rigor

A full yes was reached in 9.7% (10/103) of SRMAs in this domain of 7 items. The item with the most positive evaluation was related to the description of the risk of bias assessment in the Methods section (98/103, 95.1%), but 34.0% (35/103) of SRMA did not perform this assessment in duplicate. In addition, most of SRMAs considered only one language to study eligibility (62/103, 60.2%) and almost half did not extract data in duplicate (47/103, 45.6%) (Table 1).

### Critical appraisal

Eighteen SRMAs (17.5%) presented a full yes in this domain. A high percentage of SRMAs described the risk of bias within included studies in the Results section (89/103, 86.4%). However, only 32.0% (33/103) of SRMAs discussed the results in light of the risk of biases in individual studies. A description for non-planned modifications during the course of the review was not carried out in 17.5% (18/103) after comparing the published report to registration. Also, this comparison was not possible in 40.8% (42/103) of SRMAs due to the lack of registration (Table 1).

### Answers to SEES emails

Nine of the 103 corresponding authors questioned the assessment of some items. The median of claims was 4 items, ranging from 1 to 10. These SRMAs are identified in Additional file 7.

### Exploratory analysis

The registered SRMAs, when compared to those without registration, addressed more adequately the following items/recommended practices: availability of at least one full-search query (transparency domain) (*p* = 0.002), key eligibility criteria for study selection (completeness domain) (*p* = 0.045), detailed explanation of eligibility criteria for PICOS (completeness domain) (*p* = 0.014), statement of funding sources (completeness domain) (*p* = 0.043), potential conflicts of interest (completeness domain) (*p* = 0.043), description of the risk of bias assessment (methodological rigor domain) (*p* = 0.016), risk of bias results within studies (critical appraisal domain) (*p* = 0.000), and description for non-planned modifications (critical appraisal domain) (*p* = 0.000) (see Additional file 8). Regarding open access, we observed that 27 SRMAs had this condition and only two items, key eligibility criteria for study selection (completeness domain) (*p* = 0.049) and search in gray literature (methodological rigor) (*p* = 0.030), showed statistical significance, with greater adherence in the open access SRMAs (see Additional file 9).

## Discussion

This study presents the main report of methodological and reporting surveillance on SRMAs of physical activity intervention/exposure assessed by the SEES Initiative in the year of 2019. Across the seven domains operationalized to aggregate the items/recommended practices by the PRISMA Statement, ROBIS, and AMSTAR 2 tools, some of the most adopted practices were in the completeness domain: identification as a systematic review, meta-analysis, or both in the study title (97.1%) and more 2 items with 95.1%; outcome domain: description of the main outcome of interest in the abstract (95.1%); and methodological rigor domain: description of RoB assessment (95.1%). In opposite, the existence of a publicly available methodological protocol (4.9%) (transparency domain), discussion of the results in light of RoB in individual studies (32.0%) (critical appraisal domain), and statement on data sharing (35.9%) (transparency domain) were among the practices least adhered to, therefore indicating possible emphases that can be addressed through educational resources and editorial policies.

### Practices most and least present in assessed articles

In the transparency domain, modest existence of reviews registered in public databases and only a minimal fraction (5/103) presenting a detailed methodological protocol in addition to the registration may reduce the potential for methodological reproducibility [30]. Although most SRMAs retrieve disseminated and aggregated data, data sharing statements were still less than modest. There are similar results indicating low adherence to practices of registration [1, 24] or data sharing [31] in other disciplines, denoting that transparent practices need more implementation in varied research fields.

We observed that most studies adhered to items under the completeness, participants, and interventions/exposures domains. Taken together, these three domains included 14 items, which allow stakeholders to understand the scope of the study, the research question, its applicability, and, finally, the replication of the study if desired [21]. However, our results indicate that the information on the eligibility criteria for the selection of individual studies was not adequately reported in a relevant number of SRMAs, either in abstracts (25.2%) or in methods (31.1%). Furthermore, 22.4% of SRMAs did not provide a clear description of participants or main conditions under study in the abstract section. We underscore the importance of such information since it is key for stakeholders to decide whether to access (possibly by payment) the article.

In domains of methodological rigor, outcome reporting, and critical appraisal, we combined items (practices) that could more directly affect the interpretation of evidence generated by SRMAs. Surprisingly, several reviews did lack duplicate processes in data extraction and risk of bias assessments. Although similar results were observed in meta-research studies in health sciences [1, 24, 32], it is widely recommended that these procedures be performed by two independent researchers or primarily conducted by one researcher and fully verified by another one [22, 29]. Additionally, we found that nearly two-thirds of SRMAs (60.3%) considered only the English language to study eligibility. Such restriction is commonly applied in literature searches [33], but can introduce a systematic error, a language bias, possibly modifying meta-analytic estimates [33, 34]. In the outcome domain, items such as individual results for each analyzed study and meta-analytic summary estimates still need substantial reporting improvement to facilitate the understanding and replicability of meta-analyses. These items were considered as properly reported when all recommended elements were addressed; therefore, our assessment may have been very strict. However, to note, previous studies have also reported similar findings in the biomedical sciences [31, 35].

In the critical appraisal domain, more than 80% of our sample presented the risk of bias assessment; however, only a third (33/103) discussed their review findings in the context of potential biases identified through the assessment. Part of our results are in line with data by Buttner et al. [36], who focused only on the risk of bias assessment, and also described a high percentage of these assessments in systematic reviews and SRMAs of exercise interventions/exposures published in a single journal. Moreover, studies in biomedical literature have also reported a reduced use of this type of assessment in the discussion of SRMAs [9, 32], therefore, in agreement with our findings. In contrast, Buttner et al. [36] found more favorable results, indicating that risk of bias assessments were incorporated in the interpretation/discussion sections of 86% of their sample. Although both studies, Buttner et al. [36] and ours, have distinct samples and criteria to assess this practice, we reason that, especially when the risk of bias is high in individual studies, such results should be thoroughly addressed in Discussion sections. Finally, we assessed whether SRMAs reported methodological changes during the course of the review, which were not planned as described in the registry or methodological protocol. These changes in design, conduct, or analysis can introduce bias in the review findings and therefore should be reported to the readers’ knowledge [22, 29]. Unfortunately, our results show that 17.5% (18/103) of SRMAs made unplanned changes and did not describe them in the final publication. Additionally, in 41.7% (43/103) of SRMAs, it was not possible to carry out this verification due to the lack of a public registration record, which again reinforces the need for transparent practices at the pre-study stage.

The results observed show the importance of this type of initiative, with a characteristic of “post-publication criticism,” for the realization of a call for attention and action for the different stakeholders. It is noteworthy that the SEES Initiative could have a significant impact if authors and editors had actively engaged in the cordial dialogue about the results of their manuscript assessments, proposed by this initiative in the emails sent to these key actors. However, the reluctance to respond to criticism does not seem to be unique to the field of exercise science [37].

### Prospective registration and open access publication

The results of our exploratory analysis indicate that prospective registration is associated with better adherence to some relevant items/recommended practices in the domains of transparency, completeness, methodological rigor, and critical appraisal. To our knowledge, there are no studies on the influence of the prospective registration on the methodological and reporting standards of SRMAs in exercise sciences, but a recent study investigated this topic in systematic reviews of healthcare interventions [38]. In a similar way to our study, they evaluated a sample (*n* = 150) of systematic reviews published in 1 year (i.e., 2015) through PRISMA and AMSTAR tool. However, unlike us, they used total scores for these analyses. As main results, they observed that the total scores on PRISMA and AMSTAR were higher for those systematic reviews registered a priori, being significant only for the total scores on AMSTAR. Therefore, they concluded that prospective registration may at least indirectly improve the overall methodological quality of systematic reviews. Regarding open access, Pastorino et al. [39], evaluating only 47 systematic reviews with and without meta-analysis in the field of oncology (open access, *n* = 15 and no open access, *n* = 32), published in 2013, observed that the overall methodological and reporting standards were comparable between studies with and without open access. Similar to our study, they found a significant difference in only one item (description of the methods used to assess the risk of bias in individual studies). However, one must be aware of the large amount of statistical comparisons, which increases the probability that a difference is significant by chance. In addition, these stratified analyses were not planned. Therefore, studies specifically designed to investigate these issues (registration and open access) must be conducted to confirm these findings.

### Strengths and limitations

Several strategies exist to improve the methodological and reporting quality of SRMAs in the biomedical literature [18, 20, 24, 32, 40–42]. Our study highlights that solely relying on the availability of these documents/tools could be insufficient for adoption. In exercise sciences, there is a need for most journals to provide endorsement of the guidelines, which may trigger quality improvements [37, 38]. The use of guidelines in the peer review process has also been proposed [24, 32]. We point out that the main strength of this study is the “translational approach,” since we used established guidelines in a lively project, giving feedback to editors and authors (despite the low number of replies). In methodological grounds, we did not homogenize our assessments through a total score, allowing readers a more detailed view of adherence to items and domains. Indeed, generating such scores is not recommended, since it assumes that all items have the same weight [22]. Finally, we included some ROBIS items in our assessment form, therefore addressing more directly the methodological quality assessment of the SRMAs.

Some limitations should be considered. First, our sample of SRMAs represents only the year 2019 and only a few pre-selected scientific journals. Therefore, the results observed in our assessments are not promptly generalizable to other SRMA publications in exercise sciences. However, it seems unlikely that the quality of reporting of previous SRMAs is superior to the current ones, given that important documents providing guidance and recommendations for planning, conducting, and reporting SRMAs were published a few years ago (i.e., PRISMA in 2009, AMSTAR 2 in 2017, ROBIS in 2016); our results are in line with several studies of this nature in other health disciplines. In addition, the selected journals had features such as connection to a professional or scientific societies, considerable public reach, and impact factor. Since these features increase visibility, we believe the quality of studies is a common priority among the selected journals. Finally, unlike other studies of this kind, we proposed to assess SRMAs prospectively and systematically (i.e., monthly basis), as well as to provide direct feedback to authors and journal editors, encouraging them to question the assessments when they do not agree with these. Thus, focusing on some journals became essential to increase our chances of being able to contemplate this proposal. Second, a possible limitation is the fact that our assessment form has not been formally validated. Although the form was elaborated on the basis of a reporting guideline for systematic reviews (i.e., PRISMA) and well-planned methodological quality assessment tools (i.e., AMSTAR and ROBIS), testing the properties of our form would be important.

## Conclusion

There was a heterogeneous pattern for adherence to reporting standards proposed by the PRISMA Statement in SRMAs in exercise sciences, with structure (completeness) at a better status than practices related to transparency and full information for adequate critical appraisal. This suboptimal (sometimes low) adherence to recommended practices on quality of reporting and methodological rigor limits the reproducibility and credibility of the SRMA findings, and consequently, the evidence uptake for clinical decision-making. In other words, this can lead to an avoidable waste of resources and, ultimately, impair the application/use of the physical activity interventions investigated, the results achieved by those undergoing these interventions (patients/individuals in general), and the overall confidence in the exercise sciences. Therefore, the implementation of strategies such as SEES, which proposes to monitor published research continuously and discuss its findings openly and cordially with the authors and editors, can be a way to improve the synthesis of evidence in the field of exercise sciences. However, it seems necessary to discuss aims, outcomes, and diversify strategies to increase engagement and responsibility of all stakeholders. Finally, as future directions, we can point out that after observing the overall picture with this study, we will move on to identifying critical areas to focus on, with the few resources we have, and explore it in further publications and educational actions in social media (e.g., exploration of integrity between outcomes defined in the registry and/or protocol and the outcomes reported in the final manuscript).

## Supplementary Information

**Additional file 1.** PRISMA Checklist.

**Additional file 2.** Search strategies for journals of exercise sciences and general medicine.

**Additional file 3.** Full assessment of SRMA with individual items, associated component labels (i.e., domains) and, whether applicable, the guideline or tool of reference.

**Additional file 4.** Exceptions and operationalization of the answers of some items.

**Additional file 5: Table.** Number of Systematic Reviews with Meta-Analysis (SRMAs) assessed by journal.

**Additional file 6.** Spreadsheet with complete assessments.

**Additional file 7.** List of the studies in descending order of adherence to the items/recommended practices.

**Additional file 8: Table.** Frequency distribution of items/recommended practices by domains for the Systematic Reviews with Meta-Analysis (SRMAs) with registration and without registration.

**Additional file 9: Table.** Frequency distribution of items/recommended practices by domains for the Systematic Reviews with Meta-Analysis (SRMAs) with open access and without open access.

## Data Availability

All data generated by the SEES Initiative are publicly available via Open Science Framework (https://osf.io/ntw7d/; DOI: 10.17605/OSF.IO/NTW7D) under a CC-By Attribution 4.0 International license. Data sheets: Eligibility (https://osf.io/6jzb8/); SRMA assessments (https://osf.io/29f4a/).

## Abbreviations

ab: Abstract
AMSTAR 2: A MeaSurement Tool to Assess systematic Reviews
PICOS: Population, intervention, comparator, outcome, setting
PRISMA: Preferred Reporting Items for Systematic Reviews and Meta-Analyses Statement
RoB: Risk of bias
ROBIS: Risk of Bias in Systematic Reviews
SEES Initiative: Strengthening the Evidence in Exercise Sciences Initiative
SRMAs: Systematic reviews with meta-analysis
