# Supplementary material for: Methodological quality and reporting standards in systematic reviews with meta-analysis of physical activity studies: a report from the Strengthening the Evidence in Exercise Sciences Initiative (SEES Initiative)
Source: Syst Rev. 2021 Dec 2;10:304. doi: 10.1186/s13643-021-01845-9 (PMC8638189; doi:10.1186/s13643-021-01845-9)
Supplement: Supplementary file 2 — Additional file 2. Search strategies for journals of exercise sciences and general medicine. [file 13643_2021_1845_MOESM2_ESM.pdf]

Additional file 2

1. Search strategies for journals of exercise sciences:

MEDLINE/PubMed query for systematic review:

"Br J Sports Med"[Journal] OR "Am J Sports Med"[Journal] OR "Med Sci Sports Exerc"[Journal] OR "Eur J Prev Cardiol"[Journal] OR "Sports Med"[Journal] OR "Int J Behav Nutr Phys Act"[Journal] OR "J Physiother"[Journal] OR "J Sci Med Sport"[Journal] OR "Scand J Med Sci Sports"[Journal]

AND

(meta[tiab] OR synthesis[tiab] OR literature[tiab] OR published[tiab] OR meta-analysis[tiab] OR extraction[tiab] OR trials[tiab] OR search[tiab] OR MEDLINE[tiab] OR selection[tiab] OR sources[tiab] OR review[tiab] OR review[pt] OR articles[tiab] OR reviewed[tiab] OR english[tiab] OR language[tiab])

2. Search strategies for journals in general medicine:

MEDLINE/PubMed query for systematic review:

“Ann Intern Med”[Journal] OR “BMJ”[Journal] OR “JAMA”[Journal] OR “Lancet”[Journal] NOT ("Lancet Respir Med"[Journal] OR "Lancet Public Health"[Journal] OR "Lancet Psychiatry"[Journal] OR "Lancet Planet Health"[Journal] OR "Lancet Oncol"[Journal] OR "Lancet Neurol"[Journal] OR "Lancet Infect Dis"[Journal] OR "Lancet Haematol"[Journal] OR "Lancet HIV"[Journal] OR "Lancet Glob Health"[Journal] OR "Lancet Gastroenterol Hepatol"[Journal] OR "Lancet Diabetes Endocrinol"[Journal] OR "Lancet Child Adolesc Health"[Journal]) NOT ("Lancet Respir Med"[Journal] OR "Lancet Public Health"[Journal] OR "Lancet Psychiatry"[Journal] OR "Lancet Planet Health"[Journal] OR "Lancet Oncol"[Journal] OR "Lancet Neurol"[Journal] OR "Lancet Infect Dis"[Journal] OR "Lancet Haematol"[Journal] OR "Lancet HIV"[Journal] OR "Lancet Glob Health"[Journal] OR "Lancet Gastroenterol Hepatol"[Journal] OR "Lancet Diabetes Endocrinol"[Journal] OR "Lancet Child Adolesc Health"[Journal]) OR “N Engl J Med”[Journal]

AND

((meta[tiab] OR synthesis[tiab] OR literature[tiab] OR published[tiab] OR meta-analysis[tiab] OR

extraction[tiab] OR trials[tiab] OR search[tiab] OR MEDLINE[tiab] OR selection[tiab] OR sources[tiab] OR review[tiab] OR review[pt] OR articles[tiab] OR reviewed[tiab] OR english[tiab] OR language[tiab]) **AND (exercise[tiab] OR “physical activity”[tiab] OR training[tiab] OR rehabilitation[tiab]))**

Separated by journals:

“Ann Intern Med”[Journal] AND ((meta[tiab] OR synthesis[tiab] OR literature[tiab] OR published[tiab] OR meta-analysis[tiab] OR extraction[tiab] OR trials[tiab] OR search[tiab] OR MEDLINE[tiab] OR selection[tiab] OR sources[tiab] OR review[tiab] OR review[pt] OR articles[tiab] OR reviewed[tiab] OR english[tiab] OR language[tiab]) **AND (exercise[tiab] OR “physical activity”[tiab] OR training[tiab] OR rehabilitation[tiab]))**

“BMJ”[Journal] AND ((meta[tiab] OR synthesis[tiab] OR literature[tiab] OR published[tiab] OR meta-analysis[tiab] OR extraction[tiab] OR trials[tiab] OR search[tiab] OR MEDLINE[tiab] OR selection[tiab] OR sources[tiab] OR review[tiab] OR review[pt] OR articles[tiab] OR reviewed[tiab] OR english[tiab] OR language[tiab]) **AND (exercise[tiab] OR “physical activity”[tiab] OR training[tiab] OR rehabilitation[tiab]))**

“JAMA”[Journal] AND ((meta[tiab] OR synthesis[tiab] OR literature[tiab] OR published[tiab] OR meta-analysis[tiab] OR extraction[tiab] OR trials[tiab] OR search[tiab] OR MEDLINE[tiab] OR selection[tiab] OR sources[tiab] OR review[tiab] OR review[pt] OR articles[tiab] OR reviewed[tiab] OR english[tiab] OR language[tiab]) **AND (exercise[tiab] OR “physical activity”[tiab] OR training[tiab] OR rehabilitation[tiab]))**

“Lancet”[Journal] NOT (("Lancet Respir Med"[Journal] OR "Lancet Public Health"[Journal] OR "Lancet Psychiatry"[Journal] OR "Lancet Planet Health"[Journal] OR "Lancet Oncol"[Journal] OR "Lancet Neurol"[Journal] OR "Lancet Infect Dis"[Journal] OR "Lancet Haematol"[Journal] OR "Lancet HIV"[Journal] OR "Lancet Glob Health"[Journal] OR "Lancet Gastroenterol Hepatol"[Journal] OR "Lancet Diabetes Endocrinol"[Journal] OR "Lancet Child Adolesc Health"[Journal]) NOT ("Lancet Respir Med"[Journal] OR "Lancet Public Health"[Journal] OR "Lancet Psychiatry"[Journal] OR "Lancet Planet Health"[Journal] OR "Lancet Oncol"[Journal] OR

"Lancet Neurol"[Journal] OR "Lancet Infect Dis"[Journal] OR "Lancet Haematol"[Journal] OR "Lancet HIV"[Journal] OR "Lancet Glob Health"[Journal] OR "Lancet Gastroenterol Hepatol"[Journal] OR "Lancet Diabetes Endocrinol"[Journal] OR "Lancet Child Adolesc Health"[Journal]) AND (meta[tiab] OR synthesis[tiab] OR literature[tiab] OR published[tiab] OR meta-analysis[tiab] OR extraction[tiab] OR trials[tiab] OR search[tiab] OR MEDLINE[tiab] OR selection[tiab] OR sources[tiab] OR review[tiab] OR review[pt] OR articles[tiab] OR reviewed[tiab] OR english[tiab] OR language[tiab]) **AND (exercise[tiab] OR "physical activity"[tiab] OR training[tiab] OR rehabilitation[tiab]))**

"N Engl J Med"[Journal] AND ((meta[tiab] OR synthesis[tiab] OR literature[tiab] OR published[tiab] OR meta-analysis[tiab] OR extraction[tiab] OR trials[tiab] OR search[tiab] OR MEDLINE[tiab] OR selection[tiab] OR sources[tiab] OR review[tiab] OR review[pt] OR articles[tiab] OR reviewed[tiab] OR english[tiab] OR language[tiab]) **AND (exercise[tiab] OR "physical activity"[tiab] OR training[tiab] OR rehabilitation[tiab]))**
