# Supplementary material for: Methodological quality and reporting standards in systematic reviews with meta-analysis of physical activity studies: a report from the Strengthening the Evidence in Exercise Sciences Initiative (SEES Initiative)
Source: Syst Rev. 2021 Dec 2;10:304. doi: 10.1186/s13643-021-01845-9 (PMC8638189; doi:10.1186/s13643-021-01845-9)
Supplement: Supplementary file 3 — Additional file 3. Full assessment of SRMA with individual items, associated component labels (i.e., domains) and, whether applicable, the guideline or tool of reference. [file 13643_2021_1845_MOESM3_ESM.pdf]

**Additional file 3**

Full assessment of SRMA with individual items, associated component labels (i.e., domains) and, whether applicable, the guideline or tool of reference. The items of interest to be assessed in SRMAs were selected based on key information that is (i) suitable to be presented in systematic reviews with exercise intervention or exposures related to physical activity; (ii) relevant to allow methodological reproducibility; and (iii) important to describe how evidence is weighted in by authors in order to provide well-reasoned knowledge translation.

| **Component** | **Item** | **Reference (guideline, checklist item; unless otherwise stated)** |
|---|---|---|
| Transparency | Registration: Is the review registered in a public database? | PRISMA, 2 and 5<br>AMSTAR 2, 2 |
| Transparency | Protocol: Is there referral of a publicly available methodological protocol? (Note: if so, you must also use the protocol to consult information about the review) | PRISMA, 5<br>AMSTAR 2, 2 |
| Transparency | Methods: Is there at least one search query fully available? (Note: a full search query should allow complete replication) | PRISMA, 8 |
| Transparency | Is there a statement regarding the data availability (data sharing plan)? | Not applicable |
| Completeness | Title: Is the study identified as a systematic review, meta-analysis, or both? | PRISMA, 1 |
| Completeness | Abstract: Does the abstract list the data sources used in the review? (Note: if more than five databases were used, simplified or partial referral should be considered as "Yes") | PRISMA, 2 |
| Completeness | Abstract: Does the abstract inform key eligibility criteria for study selection? | PRISMA, 2 |

| | | |
|---|---|---|
| Completeness | Abstract: Is there a description of the number of included studies? | Not applicable |
| Completeness | Introduction: Is there a description for the research question (with PICOS elements) or precisely stated objectives (with PICOS) ? | PRISMA, 4<br>AMSTAR 2, 1 |
| Completeness | Methods: Is there a detailed explanation of eligibility criteria for PICOS elements? (Note: detailed explanation should allow complete replication) | PRISMA, 6<br>ROBIS, 1.3 |
| Completeness | Results: Is there a full description regarding the numbers of references (retrieval, eligibility, synthesis)? | PRISMA, 17 |
| Completeness | Results: Is there a description about the sample sizes of individual studies? | PRISMA, 18 |
| Completeness | Results: Is there a description of study duration (follow-up lengths)? | PRISMA, 18<br>ROBIS, 3.2 |
| Completeness | Is there a statement regarding the sources of funding? (Note: funding for the review itself) | PRISMA, 27<br>CONSORT, 25 |
| Completeness | Did the review authors declare whether they had any conflicts of interest (COI)? | AMSTAR 2, 16 |
| Participants | Abstract: Is there a description regarding the population (participants) or main condition(s) addressed in the review? | PRISMA, 2 |
| Intervention / Exposure | Abstract: Is there a description regarding the interventions/exposures (or, broadly, independent variables) addressed in the review? | PRISMA, 2 |
| Participants; Intervention / Exposure | Results: Is there a full description of characteristics? (Note: the available PICOS elements should be considered) | PRISMA, 18<br>AMSTAR 2, 8<br>ROBIS, 3.2 |
| Outcome | Abstract: Is there a result description for the main outcome of interest? | PRISMA, 2 |
| Outcome | Methods: Is there a description of the statistical combination (meta-analysis) regarding the effect measure (e.g., relative risk or mean difference), statistical method (e.g., inverse | PRISMA, 14<br>ROBIS, 3.3 |

| | | |
|---|---|---|
| | variance), and effects approach (fixed or random)? | |
| Outcome | Methods: Is there a description regarding the assessment of statistical heterogeneity? | PRISMA, 14 |
| Outcome | Results: Is there a minimally recommended description of meta-analytic summary estimates? (Note: binary outcomes as frequencies with and without the event (or as proportions such as 12/45); continuous outcomes as the mean, standard deviation, and sample size for each group) | PRISMA, 20, 21 |
| Outcome | Results: Is there a full description of individual results for studies composing the meta-analysis? (Note: effects size, imprecision measure and percentage weight should be considered) | PRISMA, 20 |
| Methodological rigor | Methods: Did the search strategy include non-published evidence? ("grey literature") | PRISMA, 7<br>AMSTAR 2, 4<br>ROBIS 2.1 |
| Methodological rigor | Methods: If the search is restricted for evidence generated after 1980, is there an indirect or direct justification related to the time range? | PRISMA, 8<br>ROBIS, 2.4 |
| Methodological rigor | Methods: How many languages were considered for study eligibility? | PRISMA, 6<br>ROBIS, 2.4 |
| Methodological rigor | Methods: Was the study selection carried out in duplicate? | PRISMA, 10<br>AMSTAR 2, 5<br>ROBIS, 2.5 |
| Methodological rigor | Methods: Was the data extraction carried out in duplicate? | PRISMA, 11<br>AMSTAR 2, 6 |
| Methodological rigor | Methods: Is there a description of the assessment of risk of biases? | PRISMA, 12<br>ROBIS, 3.4 |
| Methodological rigor | Methods: Was the assessment of risk of biases carried out in duplicate? | ROBIS, 3.5 |

| | | |
|---|---|---|
| Critical appraisal | Results: Is there a description of risk of bias within studies? (Note: your assessment should be based on the characteristic of the RoB tool) | PRISMA, 22 |
| Critical appraisal | Results: Is there a description for non-planned modifications to the synthesis during the course of the review? (e.g.: change in eligibility criteria or RoB tools; please, what was changed and its justification [why] should be considered). | AMSTAR 2, 2<br>ROBIS, 4.2 |
| Critical appraisal | Discussion: Is there a potential spin bias based on a specific reporting strategy to highlight that the experimental treatment (or condition of interest) leads to the hypothesized result? | Based on Boutron et al [1]<br>ROBIS, C (Phase 3) |
| Critical appraisal | Discussion: Are the results discussed in light of the risk of biases in individual studies? | PRISMA, 25<br>AMSTAR 2, 13<br>ROBIS, 4.6 and A (Phase 3) |
| Critical appraisal | Discussion: Are limitations discussed at the study/outcome and/or at the review level? | PRISMA, 25<br>AMSTAR 2, 12<br>ROBIS, A (Phase 3) |
| Components are coded by colors, except the item related to full description of participants and intervention (white background), which accounts twice for both Participants and Interventions/Exposures. | | |

AMSTAR: A MeaSurement Tool to Assess systematic Reviews; COI: conflict of interest; PICOS: acronym for Population, Intervention, Comparator/Control, Outcome, Setting; PRISMA: Preferred Reporting Items for Systematic Reviews and Meta-Analyses; ROBIS: Risk Of Bias In Systematic Reviews; RoB: risk of bias.

| **Component label** | **Number of items** |
|---|---|
| Transparency | 4 |
| Completeness | 11 |
| Participants | 2 |
| Intervention / Exposure | 2 |

| | |
|---|---|
| Methodological rigor | 7 |
| Outcome | 5 |
| Critical appraisal | 5 |
| Note: As a same item (relating to both Intervention/Exposure and Participants components) accounts twice, the final sum totalizes 36 items. | |

## Reference

1. Boutron I, Dutton S, Ravaud P, Altman DG. Reporting and interpretation of randomized controlled trials with statistically nonsignificant results for primary outcomes. JAMA. 2010;303:2058–64.
