# Supplementary material for: Methodological quality and reporting standards in systematic reviews with meta-analysis of physical activity studies: a report from the Strengthening the Evidence in Exercise Sciences Initiative (SEES Initiative)
Source: Syst Rev. 2021 Dec 2;10:304. doi: 10.1186/s13643-021-01845-9 (PMC8638189; doi:10.1186/s13643-021-01845-9)
Supplement: Supplementary file 4 — Additional file 4. Exceptions and operationalization of the answers of some items. [file 13643_2021_1845_MOESM4_ESM.pdf]

Additional file 4

Exceptions and operationalization of the answers of some items

Items where we considered more than one answer, the answer "No" or another answer as being "positive" (i.e., recommended practice):

1. **Methods: If the search is restricted for evidence generated after 1980, is there an indirect or direct justification related to the time range? (Methodological rigor)**
   The possibilities considered "positive" were: "*Yes*" or "*The searches were carried out from the database inception (earliest date)"*. Therefore, these two possibilities were combined as "*Yes*", when the result of this question was presented in binary form (i.e., yes or no).

2. **Methods: Was the study selection carried out in duplicate? (Methodological rigor)**
   Methods: Was the data extraction carried out in duplicate? (Methodological rigor)
   Methods: Was the assessment of risk of biases carried out in duplicate? (Methodological rigor)
   The possibilities considered "positive" were: "*Yes*" or "*Partial Yes (e.g., a sample of 50% of studies were checked by two independent researchers)*". Therefore, these two possibilities were combined as "*Yes*", when the result of this question was presented in binary form (i.e., yes or no).

3. **Results: Is there a description of risk of bias within studies? (Note: your assessment should be based on the characteristic of the RoB tool) (Critical appraisal)**
   The possibilities considered "positive" were: "*Yes (FULL description)*" or "*Partial Yes (there are individual results without specification of specific*

criteria/domains)". Therefore, these two possibilities were combined as "*Yes*", when the result of this question was presented in binary form (i.e., yes or no).

4. **Results: Is there a description for non-planned modifications to the synthesis during the course of the review? (e.g.: change in eligibility criteria or RoB tools; please, what was changed and its justification [why] should be considered) (Critical appraisal)**

   The possibilities considered "positive" were: "*Yes*" or "*Does not apply*". Here "*Does not apply*" means that after comparing the article to the register or protocol it was not identified any non-planned modification to be described in the main text. Therefore, these two possibilities were combined as "*Yes*", when the result of this question was presented in binary form (i.e., yes or no).

5. **Results: Is there a description of study duration (follow-up lengths)? Completeness**

   The possibilities considered "positive" were: "*Yes*" or "*Does not apply*". Here "*Does not apply*" was the possibility of choice when the SRMA assessed was from observational studies. Therefore, these two possibilities were combined as "*Yes*", when the result of this question was presented in binary form (i.e., yes or no).

6. **Methods: How many languages were considered for study eligibility? (Methodological rigor)**

   The possibilities considered "positive" were: "*No restriction*" or "more than 1 language". Therefore, these two possibilities were combined as "*Yes*", when the result of this question was presented in binary form (i.e., yes or no).

7. **Discussion: Is there a potential spin bias based on a specific reporting strategy to highlight that the experimental treatment (or condition of interest) leads to the hypothesized result? (Critical appraisal)**

   The possibility considered "positive" was the answer "*No*".

8. **Discussion: Are limitations discussed at the study/outcome and/or at the review level? (Critical appraisal)**

   There were three possibilities of "*Yes*": "*Yes, BOTH for study and review levels*"; "*Yes, ONLY for the review level (limitation within or across studies not mentioned)*"; "*Yes, ONLY for the review level (limitation within or across studies not mentioned)*". These three were considered as "positive".

Item where we considered more than one answer as "No":

9. **Results: Is there a description for non-planned modifications to the synthesis during the course of the review? (e.g.: change in eligibility criteria or RoB tools; please, what was changed and its justification [why] should be considered) (Critical appraisal)**

   The answer "*Unclear*" was combined with "*No*" when the result of this question was presented in binary form (i.e., yes or no). Here, "*Unclear*" was the possibility of choice when the SRMA assessed did not have a record or protocol to compare with the study report and verify the existence of non-planned modifications.
