# Supplementary material for: Methodological quality and reporting standards in systematic reviews with meta-analysis of physical activity studies: a report from the Strengthening the Evidence in Exercise Sciences Initiative (SEES Initiative)
Source: Syst Rev. 2021 Dec 2;10:304. doi: 10.1186/s13643-021-01845-9 (PMC8638189; doi:10.1186/s13643-021-01845-9)
Supplement: Supplementary file 5 — Additional file 5: Table. Number of Systematic Reviews with Meta-Analysis (SRMAs) assessed by journal. [file 13643_2021_1845_MOESM5_ESM.pdf]

**Additional file 5**

**Table. Number of Systematic Reviews with Meta-Analysis (SRMAs) assessed by journal**

| Journal | SRMAs (n) |
|---|---|
| American Journal of Sports Medicine | 4 |
| British Journal of Sports Medicine | 34 |
| European Journal of Preventive Cardiology | 6 |
| International Journal of Behavioral Nutrition and Physical Activity | 7 |
| Journal of Physiotherapy | 3 |
| Journal of Science and Medicine in Sport | 6 |
| Medicine and Science in Sports and Exercise | 1 |
| Scandinavian Journal of Medicine & Science in Sports | 5 |
| Sports Medicine | 36 |
| The British Medical Journal | 1 |
| **Total** | **103** |

Journals without SRMAs assessed: Annals of Internal Medicine, Journal of the American Medical Association, Lancet, New England Journal of Medicine
