# Supplementary material for: Methodological quality and reporting standards in systematic reviews with meta-analysis of physical activity studies: a report from the Strengthening the Evidence in Exercise Sciences Initiative (SEES Initiative)
Source: Syst Rev. 2021 Dec 2;10:304. doi: 10.1186/s13643-021-01845-9 (PMC8638189; doi:10.1186/s13643-021-01845-9)
Supplement: Supplementary file 7 — Additional file 7. List of the studies in descending order of adherence to the items/recommended practices. [file 13643_2021_1845_MOESM7_ESM.pdf]

Additional file 7

List of the studies in descending order of adherence to the items/practices recommended (*Corresponding author answered to the SEES email)

1. Efficacy of heavy eccentric calf training for treating mid-portion Achilles tendinopathy: a systematic review and meta-analysis.
   Murphy MC, Travers MJ, Chivers P, Debenham JR, Docking SI, Rio EK, Gibson W.
   Br J Sports Med. 2019 Sep;53(17):1070-1077. doi: 10.1136/bjsports-2018-099934. Epub 2019 Jan 13.
   PMID: 30636702
2. Comparative effectiveness of treatment options for plantar heel pain: a systematic review with network meta-analysis. *
   Babatunde OO, Legha A, Littlewood C, Chesterton LS, Thomas MJ, Menz HB, van der Windt D, Roddy E.
   Br J Sports Med. 2019 Feb;53(3):182-194. doi: 10.1136/bjsports-2017-098998. Epub 2018 Jun 28.
   PMID: 29954828
3. Multifactorial interventions for preventing falls in older people living in the community: a systematic review and meta-analysis of 41 trials and almost 20 000 participants.
   Hopewell S, Copsey B, Nicolson P, Adedire B, Boniface G, Lamb S.
   Br J Sports Med. 2020 Nov;54(22):1340-1350. doi: 10.1136/bjsports-2019-100732. Epub 2019 Aug 21.
   PMID: 31434659 Free PMC article.
4. Including the Nordic hamstring exercise in injury prevention programmes halves the rate of hamstring injuries: a systematic review and meta-analysis of 8459 athletes. *
   van Dyk N, Behan FP, Whiteley R.
   Br J Sports Med. 2019 Nov;53(21):1362-1370. doi: 10.1136/bjsports-2018-100045. Epub 2019 Feb 26.

PMID: 30808663

5. Effectiveness of interventions using self-monitoring to reduce sedentary behavior in adults: a systematic review and meta-analysis.

   Compernolle S, DeSmet A, Poppe L, Crombez G, De Bourdeaudhuij I, Cardon G, van der Ploeg HP, Van Dyck D.

   Int J Behav Nutr Phys Act. 2019 Aug 13;16(1):63. doi: 10.1186/s12966-019-0824-3.

   PMID: 31409357 Free PMC article.

6. A Systematic Review and Meta-Analysis of Crossover Studies Comparing Physiological, Perceptual and Performance Measures Between Treadmill and Overground Running.

   Miller JR, Van Hooren B, Bishop C, Buckley JD, Willy RW, Fuller JT.

   Sports Med. 2019 May;49(5):763-782. doi: 10.1007/s40279-019-01087-9.

   PMID: 30847825

7. Effect of interventions using physical activity trackers on physical activity in people aged 60 years and over: a systematic review and meta-analysis.

   S Oliveira J, Sherrington C, R Y Zheng E, Franco MR, Tiedemann A.

   Br J Sports Med. 2020 Oct;54(20):1188-1194. doi: 10.1136/bjsports-2018-100324. Epub 2019 Aug 9.

   PMID: 31399430

8. Is running associated with a lower risk of all-cause, cardiovascular and cancer mortality, and is the more the better? A systematic review and meta-analysis.

   Pedisic Z, Shrestha N, Kovalchik S, Stamatakis E, Liangruenrom N, Grgic J, Titze S, Biddle SJ, Bauman AE, Oja P.

   Br J Sports Med. 2020 Aug;54(15):898-905. doi: 10.1136/bjsports-2018-100493. Epub 2019 Nov 4.

   PMID: 31685526

9. Effects of Evening Exercise on Sleep in Healthy Participants: A Systematic Review and Meta-Analysis.

   Stutz J, Eiholzer R, Spengler CM.

   Sports Med. 2019 Feb;49(2):269-287. doi: 10.1007/s40279-018-1015-0.

PMID: 30374942

10. Is Muscular Fitness Associated with Future Health Benefits in Children and Adolescents? A Systematic Review and Meta-Analysis of Longitudinal Studies.
García-Hermoso A, Ramírez-Campillo R, Izquierdo M.
Sports Med. 2019 Jul;49(7):1079-1094. doi: 10.1007/s40279-019-01098-6.
PMID: 30953308

11. Aerobic exercise interventions reduce blood pressure in patients after stroke or transient ischaemic attack: a systematic review and meta-analysis.
Wang C, Redgrave J, Shafizadeh M, Majid A, Kilner K, Ali AN.
Br J Sports Med. 2019 Dec;53(24):1515-1525. doi: 10.1136/bjsports-2017-098903. Epub 2018 May 9.
PMID: 29743171

12. Effects of prenatal exercise on fetal heart rate, umbilical and uterine blood flow: a systematic review and meta-analysis.
Skow RJ, Davenport MH, Mottola MF, Davies GA, Poitras VJ, Gray CE, Jaramillo Garcia A, Barrowman N, Meah VL, Slater LG, Adamo KB, Barakat R, Ruchat SM.
Br J Sports Med. 2019 Jan;53(2):124-133. doi: 10.1136/bjsports-2018-099822. Epub 2018 Oct 18.
PMID: 30337345

13. Exercise for the prevention and treatment of low back, pelvic girdle and lumbopelvic pain during pregnancy: a systematic review and meta-analysis.
Davenport MH, Marchand AA, Mottola MF, Poitras VJ, Gray CE, Jaramillo Garcia A, Barrowman N, Sobierajski F, James M, Meah VL, Skow RJ, Riske L, Nuspl M, Nagpal TS, Courbalay A, Slater LG, Adamo KB, Davies GA, Barakat R, Ruchat SM.
Br J Sports Med. 2019 Jan;53(2):90-98. doi: 10.1136/bjsports-2018-099400. Epub 2018 Oct 18.
PMID: 30337344

14. Combined training is the most effective training modality to improve aerobic capacity and blood pressure control in people requiring haemodialysis for end-stage renal disease: systematic review and network meta-analysis.

Scapini KB, Bohlke M, Moraes OA, Rodrigues CG, Inácio JF, Sbruzzi G, Leguisamo CP, Sanches IC, Tourinho Filho H, Irigoyen MC.

J Physiother. 2019 Jan;65(1):4-15. doi: 10.1016/j.jphys.2018.11.008. Epub 2018 Dec 21.

PMID: 30581137

15. Does adding hip exercises to quadriceps exercises result in superior outcomes in pain, function and quality of life for people with knee osteoarthritis? A systematic review and meta-analysis.

    Hislop AC, Collins NJ, Tucker K, Deasy M, Semciw AI.

    Br J Sports Med. 2020 Mar;54(5):263-271. doi: 10.1136/bjsports-2018-099683. Epub 2019 Feb 6.

    PMID: 30728126

16. Aerobic exercise alleviates depressive symptoms in patients with a major non-communicable chronic disease: a systematic review and meta-analysis.

    Béland M, Lavoie KL, Briand S, White UJ, Gemme C, Bacon SL.

    Br J Sports Med. 2020 Mar;54(5):272-278. doi: 10.1136/bjsports-2018-099360. Epub 2019 Feb 6.

    PMID: 30728127

17. Cost-effectiveness of exercise therapy in the treatment of non-specific neck pain and low back pain: a systematic review with meta-analysis.

    Miyamoto GC, Lin CC, Cabral CMN, van Dongen JM, van Tulder MW.

    Br J Sports Med. 2019 Feb;53(3):172-181. doi: 10.1136/bjsports-2017-098765. Epub 2018 Apr 20.

    PMID: 29678893

18. The effects of yoga compared to active and inactive controls on physical function and health related quality of life in older adults- systematic review and meta-analysis of randomised controlled trials.

    Sivaramakrishnan D, Fitzsimons C, Kelly P, Ludwig K, Mutrie N, Saunders DH, Baker G.

    Int J Behav Nutr Phys Act. 2019 Apr 5;16(1):33. doi: 10.1186/s12966-019-0789-2.

    PMID: 30953508 Free PMC article.

19. Systematic review and meta-analysis of maintenance of physical activity behaviour change in cancer survivors.

Grimmett C, Corbett T, Brunet J, Shepherd J, Pinto BM, May CR, Foster C.

Int J Behav Nutr Phys Act. 2019 Apr 27;16(1):37. doi: 10.1186/s12966-019-0787-4.

PMID: 31029140 Free PMC article.

20. Exercise-based cardiac rehabilitation in patients with reduced left ventricular ejection fraction: The Cardiac Rehabilitation Outcome Study in Heart Failure (CROS-HF): A systematic review and meta-analysis.

Bjarnason-Wehrens B, Nebel R, Jensen K, Hackbusch M, Grilli M, Gielen S, Schwaab B, Rauch B; German Society of Cardiovascular Prevention and Rehabilitation (DGPR).

Eur J Prev Cardiol. 2020 Jun;27(9):929-952. doi: 10.1177/2047487319854140. Epub 2019 Jun 8.

PMID: 31177833 Free PMC article.

21. Association of high amounts of physical activity with mortality risk: a systematic review and meta-analysis.

Blond K, Brinkløv CF, Ried-Larsen M, Crippa A, Grøntved A.

Br J Sports Med. 2020 Oct;54(20):1195-1201. doi: 10.1136/bjsports-2018-100393. Epub 2019 Aug 12.

PMID: 31406017

22. Dose-response associations between accelerometry measured physical activity and sedentary time and all cause mortality: systematic review and harmonised meta-analysis.

Ekelund U, Tarp J, Steene-Johannessen J, Hansen BH, Jefferis B, Fagerland MW, Whincup P, Diaz KM, Hooker SP, Chernofsky A, Larson MG, Spartano N, Vasan RS, Dohrn IM, Hagströmer M, Edwardson C, Yates T, Shiroma E, Anderssen SA, Lee IM.

BMJ. 2019 Aug 21;366:l4570. doi: 10.1136/bmj.l4570.

PMID: 31434697 Free PMC article.

23. Adverse events of exercise therapy in randomised controlled trials: a systematic review and meta-analysis. *
Niemeijer A, Lund H, Stafne SN, Ipsen T, Goldschmidt CL, Jørgensen CT, Juhl CB.
Br J Sports Med. 2020 Sep;54(18):1073-1080. doi: 10.1136/bjsports-2018-100461. Epub 2019 Sep 28.
PMID: 31563884

24. Prenatal exercise is not associated with fetal mortality: a systematic review and meta-analysis.
Davenport MH, Kathol AJ, Mottola MF, Skow RJ, Meah VL, Poitras VJ, Jaramillo Garcia A, Gray CE, Barrowman N, Riske L, Sobierajski F, James M, Nagpal T, Marchand AA, Slater LG, Adamo KB, Davies GA, Barakat R, Ruchat SM.
Br J Sports Med. 2019 Jan;53(2):108-115. doi: 10.1136/bjsports-2018-099773. Epub 2018 Oct 18.
PMID: 30337346

25. Effects of different protocols of high intensity interval training for VO_2max improvements in adults: A meta-analysis of randomised controlled trials.
Wen D, Utesch T, Wu J, Robertson S, Liu J, Hu G, Chen H.
J Sci Med Sport. 2019 Aug;22(8):941-947. doi: 10.1016/j.jsams.2019.01.013. Epub 2019 Jan 29.
PMID: 30733142

26. Active Commuting and Multiple Health Outcomes: A Systematic Review and Meta-Analysis.
Dinu M, Pagliai G, Macchi C, Sofi F.
Sports Med. 2019 Mar;49(3):437-452. doi: 10.1007/s40279-018-1023-0.
PMID: 30446905

27. Role of Physical Activity and Sedentary Behavior in the Mental Health of Preschoolers, Children and Adolescents: A Systematic Review and Meta-Analysis.
Rodriguez-Ayllon M, Cadenas-Sánchez C, Estévez-López F, Muñoz NE, Mora-Gonzalez J, Migueles JH, Molina-García P, Henriksson H, Mena-Molina A, Martínez-Vizcaíno V, Catena A, Löf M, Erickson KI, Lubans DR, Ortega FB, Esteban-Cornejo I.

Sports Med. 2019 Sep;49(9):1383-1410. doi: 10.1007/s40279-019-01099-5.
PMID: 30993594

28. Exercise Frequency Determines Heart Rate Variability Gains in Older People: A Meta-Analysis and Meta-Regression.
Raffin J, Barthélémy JC, Dupré C, Pichot V, Berger M, Féasson L, Busso T, Da Costa A, Colvez A, Montuy-Coquard C, Bouvier R, Bongue B, Roche F, Hupin D.
Sports Med. 2019 May;49(5):719-729. doi: 10.1007/s40279-019-01097-7.
PMID: 30945205

29. Effects of Workplace-Based Physical Activity Interventions on Cardiorespiratory Fitness: A Systematic Review and Meta-Analysis of Controlled Trials.
Burn NL, Weston M, Maguire N, Atkinson G, Weston KL.
Sports Med. 2019 Aug;49(8):1255-1274. doi: 10.1007/s40279-019-01125-6.
PMID: 31115827

30. Cycling is associated with a lower incidence of cardiovascular diseases and death: Part 1 - systematic review of cohort studies with meta-analysis.
Nordengen S, Andersen LB, Solbraa AK, Riiser A.
Br J Sports Med. 2019 Jul;53(14):870-878. doi: 10.1136/bjsports-2018-099099. Epub 2019 May 31.
PMID: 31151937

31. Cycling and cardiovascular disease risk factors including body composition, blood lipids and cardiorespiratory fitness analysed as continuous variables: Part 2-systematic review with meta-analysis.
Nordengen S, Andersen LB, Solbraa AK, Riiser A.
Br J Sports Med. 2019 Jul;53(14):879-885. doi: 10.1136/bjsports-2018-099778. Epub 2019 May 31.
PMID: 31151938

32. The Impact of Different Types of Exercise Training on Peripheral Blood Brain-Derived Neurotrophic Factor Concentrations in Older Adults: A Meta-Analysis.
Marinus N, Hansen D, Feys P, Meesen R, Timmermans A, Spildooren J.
Sports Med. 2019 Oct;49(10):1529-1546. doi: 10.1007/s40279-019-01148-z.
PMID: 31270754

33. The Effects of Continuous Compared to Accumulated Exercise on Health: A Meta-Analytic Review. *
Murphy MH, Lahart I, Carlin A, Murtagh E.
Sports Med. 2019 Oct;49(10):1585-1607. doi: 10.1007/s40279-019-01145-2.
PMID: 31267483 Free PMC article.

34. The effect of exercise training on blood pressure in African and Asian populations: A systematic review and meta-analysis of randomized controlled trials. *
Bersaoui M, Baldew SM, Cornelis N, Toelsie J, Cornelissen VA.
Eur J Prev Cardiol. 2020 Mar;27(5):457-472. doi: 10.1177/2047487319871233. Epub 2019 Aug 26.
PMID: 31450966

35. Effects of Interrupting Prolonged Sitting with Physical Activity Breaks on Blood Glucose, Insulin and Triacylglycerol Measures: A Systematic Review and Meta-analysis.
Loh R, Stamatakis E, Folkerts D, Allgrove JE, Moir HJ.
Sports Med. 2020 Feb;50(2):295-330. doi: 10.1007/s40279-019-01183-w.
PMID: 31552570 Free PMC article.

36. Effect of Strength Training on Biomechanical and Neuromuscular Variables in Distance Runners: A Systematic Review and Meta-Analysis.
Trowell D, Vicenzino B, Saunders N, Fox A, Bonacci J.
Sports Med. 2020 Jan;50(1):133-150. doi: 10.1007/s40279-019-01184-9.
PMID: 31541409

37. Comparative efficacy of exercise and anti-hypertensive pharmacological interventions in reducing blood pressure in people with hypertension: A network meta-analysis.
Noone C, Leahy J, Morrissey EC, Newell J, Newell M, Dwyer CP, Murphy J, Doyle F, Murphy AW, Molloy GJ.
Eur J Prev Cardiol. 2020 Feb;27(3):247-255. doi: 10.1177/2047487319879786. Epub 2019 Oct 15.
PMID: 31615283

38. Running to Lower Resting Blood Pressure: A Systematic Review and Meta-analysis.
Igarashi Y, Nogami Y.
Sports Med. 2020 Mar;50(3):531-541. doi: 10.1007/s40279-019-01209-3.
PMID: 31677122 Review.

39. Effectiveness of physical activity interventions for overweight and obesity during pregnancy: a systematic review of the content of behaviour change interventions.
Flannery C, Fredrix M, Olander EK, McAuliffe FM, Byrne M, Kearney PM.
Int J Behav Nutr Phys Act. 2019 Nov 1;16(1):97. doi: 10.1186/s12966-019-0859-5.
PMID: 31675954 Free PMC article.

40. Effect of workplace physical activity interventions on the cardio-metabolic health of working adults: systematic review and meta-analysis. *
Mulchandani R, Chandrasekaran AM, Shivashankar R, Kondal D, Agrawal A, Panniyammakal J, Tandon N, Prabhakaran D, Sharma M, Goenka S.
Int J Behav Nutr Phys Act. 2019 Dec 19;16(1):134. doi: 10.1186/s12966-019-0896-0.
PMID: 31856826 Free PMC article.

41. Impact of prenatal exercise on maternal harms, labour and delivery outcomes: a systematic review and meta-analysis.
Davenport MH, Ruchat SM, Sobierajski F, Poitras VJ, Gray CE, Yoo C, Skow RJ, Jaramillo Garcia A, Barrowman N, Meah VL, Nagpal TS, Riske L, James M, Nuspl M, Weeks A, Marchand AA, Slater LG, Adamo KB, Davies GA, Barakat R, Mottola MF.
Br J Sports Med. 2019 Jan;53(2):99-107. doi: 10.1136/bjsports-2018-099821. Epub 2018 Oct 18.
PMID: 30337349

42. Effects of prenatal exercise on incidence of congenital anomalies and hyperthermia: a systematic review and meta-analysis.

Davenport MH, Yoo C, Mottola MF, Poitras VJ, Jaramillo Garcia A, Gray CE, Barrowman N, Davies GA, Kathol A, Skow RJ, Meah VL, Riske L, Sobierajski F, James M, Nagpal TS, Marchand AA, Slater LG, Adamo KB, Barakat R, Ruchat SM.
Br J Sports Med. 2019 Jan;53(2):116-123. doi: 10.1136/bjsports-2018-099653. Epub 2018 Oct 18.
PMID: 30337347

43. Aquatic exercise improves motor impairments in people with Parkinson's disease, with similar or greater benefits than land-based exercise: a systematic review.
Cugusi L, Manca A, Bergamin M, Di Blasio A, Monticone M, Deriu F, Mercuro G.
J Physiother. 2019 Apr;65(2):65-74. doi: 10.1016/j.jphys.2019.02.003. Epub 2019 Mar 21.
PMID: 30904467

44. Effects of Small-Sided Games vs. Conventional Endurance Training on Endurance Performance in Male Youth Soccer Players: A Meta-Analytical Comparison.
Moran J, Blagrove RC, Drury B, Fernandes JFT, Paxton K, Chaabene H, Ramirez-Campillo R.
Sports Med. 2019 May;49(5):731-742. doi: 10.1007/s40279-019-01086-w.
PMID: 30868441

45. Effects of training and competition on the sleep of elite athletes: a systematic review and meta-analysis.
Roberts SSH, Teo WP, Warmington SA.
Br J Sports Med. 2019 Apr;53(8):513-522. doi: 10.1136/bjsports-2018-099322. Epub 2018 Sep 14.
PMID: 30217831

46. Resistance training as a treatment for older persons with peripheral artery disease: a systematic review and meta-analysis.
Parmenter BJ, Mavros Y, Ritti Dias R, King S, Fiatarone Singh M.
Br J Sports Med. 2020 Apr;54(8):452-461. doi: 10.1136/bjsports-2018-100205. Epub 2019 Apr 12.
PMID: 30979698

47. Programme frequency, type, time and duration do not explain the effects of balance exercise in older adults: a systematic review with a meta-regression analysis.

Farlie MK, Robins L, Haas R, Keating JL, Molloy E, Haines TP.

Br J Sports Med. 2019 Aug;53(16):996-1002. doi: 10.1136/bjsports-2016-096874. Epub 2018 Jan 25.

PMID: 29371222

48. The Effect of Low-Volume High-Intensity Interval Training on Body Composition and Cardiorespiratory Fitness: A Systematic Review and Meta-Analysis.

Sultana RN, Sabag A, Keating SE, Johnson NA.

Sports Med. 2019 Nov;49(11):1687-1721. doi: 10.1007/s40279-019-01167-w.

PMID: 31401727

49. Effects of Physical Exercise Training in the Workplace on Physical Fitness: A Systematic Review and Meta-analysis.

Prieske O, Dalager T, Herz M, Hortobagyi T, Sjøgaard G, Søgaard K, Granacher U.

Sports Med. 2019 Dec;49(12):1903-1921. doi: 10.1007/s40279-019-01179-6.

PMID: 31494864

50. Exercise alone and exercise combined with education both prevent episodes of low back pain and related absenteeism: systematic review and network meta-analysis of randomised controlled trials (RCTs) aimed at preventing back pain.

Huang R, Ning J, Chuter VH, Taylor JB, Christophe D, Meng Z, Xu Y, Jiang L.

Br J Sports Med. 2020 Jul;54(13):766-770. doi: 10.1136/bjsports-2018-100035. Epub 2019 Oct 31.

PMID: 31672696

51. Physically active lessons in schools and their impact on physical activity, educational, health and cognition outcomes: a systematic review and meta-analysis.

Norris E, van Steen T, Direito A, Stamatakis E.

Br J Sports Med. 2020 Jul;54(14):826-838. doi: 10.1136/bjsports-2018-100502. Epub 2019 Oct 16.

PMID: 31619381

52. A systematic review and meta-analysis of the overall effects of school-based obesity prevention interventions and effect differences by intervention components.

Liu Z, Xu HM, Wen LM, Peng YZ, Lin LZ, Zhou S, Li WH, Wang HJ.

Int J Behav Nutr Phys Act. 2019 Oct 29;16(1):95. doi: 10.1186/s12966-019-0848-8.

PMID: 31665040 Free PMC article.

53. Acute Effects of Foam Rolling on Range of Motion in Healthy Adults: A Systematic Review with Multilevel Meta-analysis. *

Wilke J, Müller AL, Giesche F, Power G, Ahmedi H, Behm DG.

Sports Med. 2020 Feb;50(2):387-402. doi: 10.1007/s40279-019-01205-7.

PMID: 31628662

54. The Effects of Interval and Continuous Training on the Oxygen Cost of Running in Recreational Runners: A Systematic Review and Meta-analysis.

González-Mohíno F, Santos-Concejero J, Yustres I, González-Ravé JM.

Sports Med. 2020 Feb;50(2):283-294. doi: 10.1007/s40279-019-01201-x.

PMID: 31606879

55. What is the effect of aerobic exercise intensity on cardiorespiratory fitness in those undergoing cardiac rehabilitation? A systematic review with meta-analysis.

Mitchell BL, Lock MJ, Davison K, Parfitt G, Buckley JP, Eston RG.

Br J Sports Med. 2019 Nov;53(21):1341-1351. doi: 10.1136/bjsports-2018-099153. Epub 2018 Aug 18.

PMID: 30121584

56. Are we missing the target? Are we aiming too low? What are the aerobic exercise prescriptions and their effects on markers of cardiovascular health and systemic inflammation in patients with knee osteoarthritis? A systematic review and meta-analysis.

Schulz JM, Birmingham TB, Atkinson HF, Woehrle E, Primeau CA, Lukacs MJ, Al-Khazraji BK, Khan MCM, Zomar BO, Petrella RJ, Beier F, Appleton CT, Shoemaker JK, Bryant DM.

Br J Sports Med. 2020 Jul;54(13):771-775. doi: 10.1136/bjsports-2018-100231. Epub 2019 Dec 17.

PMID: 31848152

57. Additional effects of psychological interventions on subjective and objective outcomes compared with exercise-based cardiac rehabilitation alone in patients with cardiovascular disease: A systematic review and meta-analysis.

Albus C, Herrmann-Lingen C, Jensen K, Hackbusch M, Münch N, Kuncewicz C, Grilli M, Schwaab B, Rauch B; German Society of Cardiovascular Prevention & Rehabilitation (DGPR).

Eur J Prev Cardiol. 2019 Jul;26(10):1035-1049. doi: 10.1177/2047487319832393. Epub 2019 Mar 11.

PMID: 30857429 Free PMC article.

58. Relative Efficacy of Different Exercises for Pain, Function, Performance and Quality of Life in Knee and Hip Osteoarthritis: Systematic Review and Network Meta-Analysis.

Goh SL, Persson MSM, Stocks J, Hou Y, Welton NJ, Lin J, Hall MC, Doherty M, Zhang W.

Sports Med. 2019 May;49(5):743-761. doi: 10.1007/s40279-019-01082-0.

PMID: 30830561 Free PMC article.

59. The Acute Neuromuscular Responses to Cluster Set Resistance Training: A Systematic Review and Meta-Analysis. *

Latella C, Teo WP, Drinkwater EJ, Kendall K, Haff GG.

Sports Med. 2019 Dec;49(12):1861-1877. doi: 10.1007/s40279-019-01172-z.

PMID: 31506904 Free PMC article.

60. Effectiveness of interventions for reducing non-occupational sedentary behaviour in adults and older adults: a systematic review and meta-analysis.

Shrestha N, Grgic J, Wiesner G, Parker A, Podnar H, Bennie JA, Biddle SJH, Pedisic Z.

Br J Sports Med. 2019 Oct;53(19):1206-1213. doi: 10.1136/bjsports-2017-098270. Epub 2018 Jan 13.

PMID: 29331992

61. Effects of Blood Flow Restriction Training on Muscular Strength and Hypertrophy in Older Individuals: A Systematic Review and Meta-Analysis.
Centner C, Wiegel P, Gollhofer A, König D.
Sports Med. 2019 Jan;49(1):95-108. doi: 10.1007/s40279-018-0994-1.
PMID: 30306467 Free PMC article.

62. Effects of chronic exercise interventions on executive function among children and adolescents: a systematic review with meta-analysis.
Xue Y, Yang Y, Huang T.
Br J Sports Med. 2019 Nov;53(22):1397-1404. doi: 10.1136/bjsports-2018-099825. Epub 2019 Feb 8.
PMID: 30737201

63. Aerobic Training Protects Cardiac Function During Advancing Age: A Meta-Analysis of Four Decades of Controlled Studies.
Beaumont AJ, Grace FM, Richards JC, Campbell AK, Sculthorpe NF.
Sports Med. 2019 Feb;49(2):199-219. doi: 10.1007/s40279-018-1004-3.
PMID: 30374946 Free PMC article.

64. How does light-intensity physical activity associate with adult cardiometabolic health and mortality? Systematic review with meta-analysis of experimental and observational studies.
Chastin SFM, De Craemer M, De Cocker K, Powell L, Van Cauwenberg J, Dall P, Hamer M, Stamatakis E.
Br J Sports Med. 2019 Mar;53(6):370-376. doi: 10.1136/bjsports-2017-097563. Epub 2018 Apr 25.
PMID: 29695511 Free PMC article.

65. The association of resistance training with mortality: A systematic review and meta-analysis.
Saeidifard F, Medina-Inojosa JR, West CP, Olson TP, Somers VK, Bonikowske AR, Prokop LJ, Vinciguerra M, Lopez-Jimenez F.
Eur J Prev Cardiol. 2019 Oct;26(15):1647-1665. doi: 10.1177/2047487319850718. Epub 2019 May 19.
PMID: 31104484

66. The Effect of Resistance Training in Women on Dynamic Strength and Muscular Hypertrophy: A Systematic Review with Meta-analysis.
Hagstrom AD, Marshall PW, Halaki M, Hackett DA.
Sports Med. 2020 Jun;50(6):1075-1093. doi: 10.1007/s40279-019-01247-x.
PMID: 31820374 Review.

67. Is interval training the magic bullet for fat loss? A systematic review and meta-analysis comparing moderate-intensity continuous training with high-intensity interval training (HIIT).
Viana RB, Naves JPA, Coswig VS, de Lira CAB, Steele J, Fisher JP, Gentil P.
Br J Sports Med. 2019 May;53(10):655-664. doi: 10.1136/bjsports-2018-099928. Epub 2019 Feb 14.
PMID: 30765340

68. Financial incentives for physical activity in adults: systematic review and meta-analysis.
Mitchell MS, Orstad SL, Biswas A, Oh PI, Jay M, Pakosh MT, Faulkner G.
Br J Sports Med. 2020 Nov;54(21):1259-1268. doi: 10.1136/bjsports-2019-100633. Epub 2019 May 15.
PMID: 31092399

69. Broad-spectrum physical fitness benefits of recreational football: a systematic review and meta-analysis.
Milanović Z, Pantelić S, Čović N, Sporiš G, Mohr M, Krustrup P.
Br J Sports Med. 2019 Aug;53(15):926-939. doi: 10.1136/bjsports-2017-097885. Epub 2018 Jan 25.
PMID: 29371223 Free PMC article.

70. Effects of Exercise Intervention on Health-Related Physical Fitness and Blood Pressure in Preschool Children: A Systematic Review and Meta-Analysis of Randomized Controlled Trials.
García-Hermoso A, Alonso-Martinez AM, Ramírez-Vélez R, Izquierdo M.
Sports Med. 2020 Jan;50(1):187-203. doi: 10.1007/s40279-019-01191-w.
PMID: 31556009

71. What are the Benefits and Risks Associated with Changing Foot Strike Pattern During Running? A Systematic Review and Meta-analysis of Injury, Running Economy, and Biomechanics.
Anderson LM, Bonanno DR, Hart HF, Barton CJ.
Sports Med. 2020 May;50(5):885-917. doi: 10.1007/s40279-019-01238-y.
PMID: 31823338 Review.

72. The Minimum Effective Training Dose Required to Increase 1RM Strength in Resistance-Trained Men: A Systematic Review and Meta-Analysis.
Androulakis-Korakakis P, Fisher JP, Steele J.
Sports Med. 2020 Apr;50(4):751-765. doi: 10.1007/s40279-019-01236-0.
PMID: 31797219 Review.

73. Revised Approach to the Role of Fatigue in Anterior Cruciate Ligament Injury Prevention: A Systematic Review with Meta-Analyses.
Benjaminse A, Webster KE, Kimp A, Meijer M, Gokeler A.
Sports Med. 2019 Apr;49(4):565-586. doi: 10.1007/s40279-019-01052-6.
PMID: 30659497 Free PMC article.

74. Exercise Dose and Weight Loss in Adolescents with Overweight-Obesity: A Meta-Regression.
Stoner L, Beets MW, Brazendale K, Moore JB, Weaver RG.
Sports Med. 2019 Jan;49(1):83-94. doi: 10.1007/s40279-018-01040-2.
PMID: 30560421

75. Does Aerobic Training Promote the Same Skeletal Muscle Hypertrophy as Resistance Training? A Systematic Review and Meta-Analysis.
Grgic J, Mcllvenna LC, Fyfe JJ, Sabol F, Bishop DJ, Schoenfeld BJ, Pedisic Z.
Sports Med. 2019 Feb;49(2):233-254. doi: 10.1007/s40279-018-1008-z.
PMID: 30341595

76. Acute Effects of Citrulline Supplementation on High-Intensity Strength and Power Performance: A Systematic Review and Meta-Analysis.
Trexler ET, Persky AM, Ryan ED, Schwartz TA, Stoner L, Smith-Ryan AE.
Sports Med. 2019 May;49(5):707-718. doi: 10.1007/s40279-019-01091-z.
PMID: 30895562

77. Effects of plyometric training on jumping, sprint performance, and lower body muscle strength in healthy adults: A systematic review and meta-analyses.

Oxfeldt M, Overgaard K, Hvid LG, Dalgas U.

Scand J Med Sci Sports. 2019 Oct;29(10):1453-1465. doi: 10.1111/sms.13487. Epub 2019 Jun 22.

PMID: 31136014

78. RETRACTED ARTICLE: Determining the Sites of Neural Adaptations to Resistance Training: A Systematic Review and Meta-Analysis.

Siddique U, Rahman S, Frazer AK, Howatson G, Kidgell DJ.

Sports Med. 2019 Nov;49(11):1809. doi: 10.1007/s40279-019-01152-3.

PMID: 31359349 Review. No abstract available.

79. Efficacy of School-Based Interventions for Improving Muscular Fitness Outcomes in Adolescent Boys: A Systematic Review and Meta-analysis.

Cox A, Fairclough SJ, Kosteli MC, Noonan RJ.

Sports Med. 2020 Mar;50(3):543-560. doi: 10.1007/s40279-019-01215-5.

PMID: 31729638 Free PMC article. Review.

80. Effects of school-based physical activity on mathematics performance in children: a systematic review. *

Sneck S, Viholainen H, Syväoja H, Kankaapää A, Hakonen H, Poikkeus AM, Tammelin T.

Int J Behav Nutr Phys Act. 2019 Nov 21;16(1):109. doi: 10.1186/s12966-019-0866-6.

PMID: 31752903 Free PMC article.

81. Exercise for preventing falls in older people living in the community: an abridged Cochrane systematic review.

Sherrington C, Fairhall N, Wallbank G, Tiedemann A, Michaleff ZA, Howard K, Clemson L, Hopewell S, Lamb S.

Br J Sports Med. 2020 Aug;54(15):885-891. doi: 10.1136/bjsports-2019-101512. Epub 2019 Dec 2.

PMID: 31792067

82. Do multi-ingredient protein supplements augment resistance training-induced gains in skeletal muscle mass and strength? A systematic review and meta-analysis of 35 trials.

O'Bryan KR, Doering TM, Morton RW, Coffey VG, Phillips SM, Cox GR.

Br J Sports Med. 2020 May;54(10):573-581. doi: 10.1136/bjsports-2018-099889. Epub 2019 Mar 1.

PMID: 30824459

83. Which exercise prescriptions optimize V̇O_2 max during cancer treatment?-A systematic review and meta-analysis.

Bjørke ACH, Sweegers MG, Buffart LM, Raastad T, Nygren P, Berntsen S.

Scand J Med Sci Sports. 2019 Sep;29(9):1274-1287. doi: 10.1111/sms.13442. Epub 2019 May 28.

PMID: 31034665

84. A Majority of Anterior Cruciate Ligament Injuries Can Be Prevented by Injury Prevention Programs: A Systematic Review of Randomized Controlled Trials and Cluster-Randomized Controlled Trials With Meta-analysis.

Huang YL, Jung J, Mulligan CMS, Oh J, Norcross MF.

Am J Sports Med. 2020 May;48(6):1505-1515. doi: 10.1177/0363546519870175. Epub 2019 Aug 30.

PMID: 31469584

85. Evaluation of school-based interventions of active breaks in primary schools: A systematic review and meta-analysis.

Masini A, Marini S, Gori D, Leoni E, Rochira A, Dallolio L.

J Sci Med Sport. 2020 Apr;23(4):377-384. doi: 10.1016/j.jsams.2019.10.008. Epub 2019 Oct 18.

PMID: 31722840

86. The effect of high Intensity interval training versus moderate intensity continuous training on arterial stiffness and 24h blood pressure responses: A systematic review and meta-analysis.

Way KL, Sultana RN, Sabag A, Baker MK, Johnson NA.

J Sci Med Sport. 2019 Apr;22(4):385-391. doi: 10.1016/j.jsams.2018.09.228. Epub 2018 Sep 22.

PMID: 30803498

87. Isolated ingestion of caffeine and sodium bicarbonate on repeated sprint performance: A systematic review and meta-analysis.

Lopes-Silva JP, Choo HC, Franchini E, Abbiss CR.

J Sci Med Sport. 2019 Aug;22(8):962-972. doi: 10.1016/j.jsams.2019.03.007. Epub 2019 Mar 20.

PMID: 31036532

88. Evidence-Based Best-Practice Guidelines for Preventing Anterior Cruciate Ligament Injuries in Young Female Athletes: A Systematic Review and Meta-analysis. *

Petushek EJ, Sugimoto D, Stoolmiller M, Smith G, Myer GD.

Am J Sports Med. 2019 Jun;47(7):1744-1753. doi: 10.1177/0363546518782460. Epub 2018 Jul 12.

PMID: 30001501 Free PMC article.

89. The Effect of Nordic Hamstring Exercise Intervention Volume on Eccentric Strength and Muscle Architecture Adaptations: A Systematic Review and Meta-analyses.

Cuthbert M, Ripley N, McMahon JJ, Evans M, Haff GG, Comfort P.

Sports Med. 2020 Jan;50(1):83-99. doi: 10.1007/s40279-019-01178-7.

PMID: 31502142 Free PMC article.

90. Safety and efficacy of aerobic exercise commenced early after cardiac surgery: A systematic review and meta-analysis.

Doyle MP, Indraratna P, Tardo DT, Peeceeyen SC, Peoples GE.

Eur J Prev Cardiol. 2019 Jan;26(1):36-45. doi: 10.1177/2047487318798924. Epub 2018 Sep 6.

PMID: 30188177

91. Combining higher-load and lower-load resistance training exercises: A systematic review and meta-analysis of findings from complex training studies.

Bauer P, Uebellacker F, Mitter B, Aigner AJ, Hasenoehrl T, Ristl R, Tschan H, Seitz LB.
J Sci Med Sport. 2019 Jul;22(7):838-851. doi: 10.1016/j.jsams.2019.01.006. Epub 2019 Jan 16.
PMID: 30683485

92. Establishing a relationship between the effect of caffeine and duration of endurance athletic time trial events: A systematic review and meta-analysis.
Shen JG, Brooks MB, Cincotta J, Manjourides JD.
J Sci Med Sport. 2019 Feb;22(2):232-238. doi: 10.1016/j.jsams.2018.07.022. Epub 2018 Aug 20.
PMID: 30170953

93. Return to Sport After Arthroscopic Rotator Cuff Repair: Is There a Difference Between the Recreational and the Competitive Athlete?
Altintas B, Anderson N, Dornan GJ, Boykin RE, Logan C, Millett PJ.
Am J Sports Med. 2020 Jan;48(1):252-261. doi: 10.1177/0363546519825624. Epub 2019 Mar 11.
PMID: 30855979

94. Impact of Pre-exercise Hypohydration on Aerobic Exercise Performance, Peak Oxygen Consumption and Oxygen Consumption at Lactate Threshold: A Systematic Review with Meta-analysis.
Deshayes TA, Jeker D, Goulet EDB.
Sports Med. 2020 Mar;50(3):581-596. doi: 10.1007/s40279-019-01223-5.
PMID: 31728846 Review.

95. Acute Effects of Resistance Exercise on Cognitive Function in Healthy Adults: A Systematic Review with Multilevel Meta-Analysis.
Wilke J, Giesche F, Klier K, Vogt L, Herrmann E, Banzer W.
Sports Med. 2019 Jun;49(6):905-916. doi: 10.1007/s40279-019-01085-x.
PMID: 30838520

96. DNA Damage Following Acute Aerobic Exercise: A Systematic Review and Meta-analysis.
Tryfidou DV, McClean C, Nikolaidis MG, Davison GW.

Sports Med. 2020 Jan;50(1):103-127. doi: 10.1007/s40279-019-01181-y.
PMID: 31529301 Free PMC article.

97. Physical exercise attenuates cognitive decline and reduces behavioural problems in people with mild cognitive impairment and dementia: a systematic review.
Law CK, Lam FM, Chung RC, Pang MY.
J Physiother. 2020 Jan;66(1):9-18. doi: 10.1016/j.jphys.2019.11.014. Epub 2019 Dec 13.
PMID: 31843427

98. Nonsurgical Treatments of Patellar Tendinopathy: Multiple Injections of Platelet-Rich Plasma Are a Suitable Option: A Systematic Review and Meta-analysis.
Andriolo L, Altamura SA, Reale D, Candrian C, Zaffagnini S, Filardo G.
Am J Sports Med. 2019 Mar;47(4):1001-1018. doi: 10.1177/0363546518759674. Epub 2018 Mar 30.
PMID: 29601207

99. The ability of exercise to meaningfully improve glucose tolerance in people living with prediabetes: A meta-analysis.
Hrubeniuk TJ, Bouchard DR, Goulet EDB, Gurd B, Sénéchal M.
Scand J Med Sci Sports. 2020 Feb;30(2):209-216. doi: 10.1111/sms.13567. Epub 2019 Oct 24.
PMID: 31593613

100. Left Ventricular Function and Cardiac Biomarker Release-The Influence of Exercise Intensity, Duration and Mode: A Systematic Review and Meta-Analysis.
Donaldson JA, Wiles JD, Coleman DA, Papadakis M, Sharma R, O'Driscoll JM.
Sports Med. 2019 Aug;49(8):1275-1289. doi: 10.1007/s40279-019-01142-5.
PMID: 31214979

101. Cooling during exercise enhances performances, but the cooled body areas matter: A systematic review with meta-analyses.
Douzi W, Dugué B, Vinches L, Al Sayed C, Hallé S, Bosquet L, Dupuy O.
Scand J Med Sci Sports. 2019 Nov;29(11):1660-1676. doi: 10.1111/sms.13521. Epub 2019 Aug 14.
PMID: 31340407

102. Metabolic Costs of Standing and Walking in Healthy Military-Age Adults: A Meta-regression.

Looney DP, Potter AW, Pryor JL, Bremner PE, Chalmers CR, McClung HL, Welles AP, Santee WR.

Med Sci Sports Exerc. 2019 Feb;51(2):346-351. doi: 10.1249/MSS.0000000000001779.

PMID: 30649093

103. The effects of exercise interventions on health and fitness of firefighters: A meta-analysis.

Andrews KL, Gallagher S, Herring MP.

Scand J Med Sci Sports. 2019 Jun;29(6):780-790. doi: 10.1111/sms.13411. Epub 2019 Mar 10.

PMID: 30779389
