# Supplementary material for: Methodological quality and reporting standards in systematic reviews with meta-analysis of physical activity studies: a report from the Strengthening the Evidence in Exercise Sciences Initiative (SEES Initiative)
Source: Syst Rev. 2021 Dec 2;10:304. doi: 10.1186/s13643-021-01845-9 (PMC8638189; doi:10.1186/s13643-021-01845-9)
Supplement: Supplementary file 8 — Additional file 8: Table. Frequency distribution of items/recommended practices by domains for the Systematic Reviews with Meta-Analysis (SRMAs) with registration and without registration. [file 13643_2021_1845_MOESM8_ESM.pdf]

Table. Frequency distribution of items/recommended practices by domains for the Systematic Reviews with Meta-Analysis (SRMAs) with registration and without registration

	SRMAs (n=103)		
	**Registration (n=57)**	**No registration (n=46)**	***p***
**Domain: Transparency**			
Protocol			0.377
No	53 (93.0%)	45 (97.8%)	
Yes	4 (7.0%)	1 (2.2%)	
Available searches			0.002*
No	5 (8.8%)	15 (32.6%)	
Yes	52 (91.2%)	31 (67.4%)	
Data Statement			0.145
No	33 (57.9%)	33 (71.7%)	
Yes	24 (42.1%)	13 (28.3%)	
**Domain: Completeness**			
Title as SRMA			0.086
No	-	3 (6.5%)	
Yes	57 (100%)	43 (93.5%)	
Data sources (ab)			0.911
No	18 (31.6%)	15 (32.6%)	
Yes	39 (68.4%)	31 (67.4%)	

Key eligibility criteria (ab)			0.045*
No	10 (17.5%)	16 (34.8%)	
Yes	47 (82.5%)	30 (65.2%)	
Number of included studies (ab)			0.654
No	2 (3.5%)	3 (6.5%)	
Yes	55 (96.5%)	43 (93.5%)	
Research question			0.644
No	15 (26.3%)	14 (30.4%)	
Yes	42 (73.7%)	32 (69.6%)	
PICOS explanation			0.014*
No	12 (21.1%)	20 (43.5%)	
Yes	45 (78.9%)	26 (56.5%)	
Number of references			0.403
No	2 (3.5%)	4 (8.7%)	
Yes	55 (96.5%)	42 (91.3%)	
Description of sample sizes			1.000
No	3 (5.3%)	2 (4.3%)	
Yes	54 (94.7%)	44 (95.7%)	
Duration of included studies			0.456
No	5 (8.8%)	2 (4.3%)	
Yes	52 (91.2%)	44 (95.7%)	
Sources of funding			0.043*
No	1 (1.8%)	6 (13.0%)	

Yes	56 (98.2%)	40 (87.0%)	
Potential conflicts of interest			0.043*
No	1 (1.8%)	6 (13.0%)	
Yes	56 (98.2%)	40 (87.0%)	
**Domain: Participants**			
Description of participants (ab)			0.125
No	9 (15.8%)	13 (28.3%)	
Yes	48 (84.2%)	33 (71.7%)	
Detailed studies' characteristics			0.064
No	5 (8.8%)	10 (21.7%)	
Yes	52 (91.2%)	36 (78.3%)	
**Domain: Intervention/exposure**			
Description of interventions/exposures (ab)			0.697
No	3 (5.3%)	4 (8.7%)	
Yes	54 (94.7%)	42 (91.3%)	
Detailed studies' characteristics			0.064
No	5 (8.8%)	10 (21.7%)	
Yes	52 (91.2%)	36 (78.3%)	
**Domain: Outcome**			
Main outcome of interest (ab)			0.170
No	1 (1.8%)	4 (8.7%)	
Yes	56 (98.2%)	42 (91.3%)	

Statistical methods			0.984
No	10 (17.5%)	8 (17.4%)	
Yes	47 (82.5%)	38 (82.6%)	
Statistical heterogeneity			0.692
No	6 (10.5%)	6 (13.0%)	
Yes	51 (89.5%)	40 (87.0%)	
Meta-analytic summary estimates			0.073
No	13 (22.8%)	18 (39.1%)	
Yes	44 (77.2%)	28 (60.9%)	
Statistics per study			0.327
No	23 (40.4%)	23 (50.0%)	
Yes	34 (59.6%)	23 (50.0%)	
**Domain: Methodological rigor**			
Searches in grey literature			0.102
No	23 (40.4%)	26 (56.5%)	
Yes	34 (59.6%)	20 (43.5%)	
Searches from inception or with justification			0.462
No	3 (5.3%)	5 (10.9%)	
Yes	54 (94.7%)	41 (89.1%)	
Number of languages			0.222
No (no statement and 1)	33 (57.9%)	32 (69.6%)	
Yes (2, 3, 4 and no restriction)	24 (42.1%)	14 (30.4%)	

Study selection in duplicate			0.276
No	12 (21.1%)	14 (30.4%)	
Yes and partial yes	45 (78.9%)	32 (69.6%)	
Data extraction in duplicate			0.234
No	29 (50.9%)	18 (39.1%)	
Yes and partial yes	28 (49.1%)	28 (60.9%)	
Description of RoB assessment			0.016*
No	-	5 (10.9%)	
Yes	57 (100%)	41 (89.1%)	
RoB assessment in duplicate			0.159
No	16 (28.1%)	19 (41.3%)	
Yes	41 (71.9%)	27 (58.7%)	
**Domain: Critical appraisal**			
RoB results within studies			0.000*
No	1 (1.8%)	13 (28.3%)	
Yes	56 (98.2%)	33 (71.7%)	
Description of protocol deviations			0.000*
No and unclear	19 (33.3%)	42 (91.3%)	
Yes and does not apply	38 (66.7%)	4 (8.7%)	
Presence of spin bias			0.075
No	49 (86.0%)	33 (71.7%)	
Yes	8 (14.0%)	13 (28.3%)	
Discussion addressing RoB			0.460

No	37 (64.9%)	33 (71.7%)	
Yes	20 (35.1%)	13 (28.3%)	
Limitations thoroughly addressed			1.000
No	3 (5.3%)	3 (6.5%)	
Yes (both for study and review levels, only for study/outcome, only for review)	54 (94.7%)	43 (93.5%)	

ab: abstract; PICOS: acronym for Population, Intervention, Comparator/Control, Outcome, Setting; RoB : risk of bias.

*$p < 0.05$
