# Supplementary material for: Methodological quality and reporting standards in systematic reviews with meta-analysis of physical activity studies: a report from the Strengthening the Evidence in Exercise Sciences Initiative (SEES Initiative)
Source: Syst Rev. 2021 Dec 2;10:304. doi: 10.1186/s13643-021-01845-9 (PMC8638189; doi:10.1186/s13643-021-01845-9)
Supplement: Supplementary file 9 — Additional file 9: Table. Frequency distribution of items/recommended practices by domains for the Systematic Reviews with Meta-Analysis (SRMAs) with open access and without open access. [file 13643_2021_1845_MOESM9_ESM.pdf]

Table. Frequency distribution of items/recommended practices by domains for the Systematic Reviews with Meta-Analysis (SRMAs) with open access and without open access

	SRMAs (n=103)		
	**Open access (n=27)**	**No Open access (n=76)**	***p***
**Domain: Transparency**			
Registration			0.633
No	11 (40.7%)	35 (46.1%)	
Yes	16 (59.3%)	41 (53.9%)	
Protocol			1.000
No	26 (96.3%)	72 (94.7%)	
Yes	1 (3.7%)	4 (5.3%)	
Available searches			0.482
No	4 (14.8%)	16 (21.1%)	
Yes	23 (85.2%)	60 (78.9%)	
Data Statement			0.123
No	14 (51.9%)	52 (68.4%)	
Yes	13 (48.1%)	24 (31.6%)	
**Domain: Completeness**			
Title as SRMA			0.565
No	-	3 (3.9%)	
Yes	27 (100%)	73 (96.1%)	

Data sources (ab)			0.517
No	10 (37.0%)	23 (30.3%)	
Yes	17 (63.0%)	53 (69.7%)	
Key eligibility criteria (ab)			0.049*
No	3 (11.1%)	23 (30.3%)	
Yes	24 (88.9%)	53 (69.7%)	
Number of included studies (ab)			0.322
No	-	5 (6.6%)	
Yes	27 (100%)	71 (93.4%)	
Research question			0.425
No	6 (22.2%)	23 (30.3%)	
Yes	21 (77.8%)	53 (69.7%)	
PICOS explanation			0.851
No	8 (29.6%)	24 (31.6%)	
Yes	19 (70.4%)	52 (68.4%)	
Number of references			0.183
No	3 (11.1%)	3 (3.9%)	
Yes	24 (88.9%)	73 (96.1%)	
Description of sample sizes			0.322
No	-	5 (6.6%)	
Yes	27 (100%)	71 (93.4%)	
Duration of included studies			1.000
No	2 (7.4%)	5 (6.6%)	

Yes	25 (92.6%)	71 (93.4%)	
Sources of funding			0.185
No	-	7 (9.2%)	
Yes	27 (100%)	69 (90.8%)	
Potential conflicts of interest			0.185
No	-	7 (9.2%)	
Yes	27 (100%)	69 (90.8%)	
**Domain: Participants**			
Description of participants (ab)			0.899
No	6 (22.2%)	16 (21.1%)	
Yes	21 (77.8%)	60 (78.9%)	
Detailed studies' characteristics			0.754
No	3 (11.1%)	12 (15.8%)	
Yes	24 (88.9%)	64 (84.2%)	
**Domain: Intervention/exposure**			
Description of interventions/exposures (ab)			0.673
No	1 (3.7%)	6 (7.9%)	
Yes	26 (96.3%)	70 (92.1%)	
Detailed studies' characteristics			0.754
No	3 (11.1%)	12 (15.8%)	
Yes	24 (88.9%)	64 (84.2%)	
**Domain: Outcome**			

Main outcome of interest (ab)			1.000
No	1 (3.7%)	4 (5.3%)	
Yes	26 (96.3%)	72 (94.7%)	
Statistical methods			0.775
No	4 (14.8%)	14 (18.4%)	
Yes	23 (85.2%)	62 (81.6%)	
Statistical heterogeneity			0.292
No	5 (18.5%)	7 (9.2%)	
Yes	22 (81.5%)	69 (90.8%)	
Meta-analytic summary estimates			0.670
No	9 (33.3%)	22 (28.9%)	
Yes	18 (66.7%)	54 (71.1%)	
Statistics per study			0.633
No	11 (40.7%)	35 (46.1%)	
Yes	16 (59.3%)	41 (53.9%)	
**Domain: Methodological rigor**			
Searches in grey literature			0.030*
No	8 (29.6%)	41 (53.9%)	
Yes	19 (70.4%)	35 (46.1%)	
Searches from inception or with justification			0.429
No	3 (11.1%)	5 (6.6%)	
Yes	24 (88.9%)	71 (93.4%)	

Number of languages			0.169
No (no statement and 1)	20 (74.1%)	45 (59.2%)	
Yes (2, 3, 4 and no restriction)	7 (25.9%)	31 (40.8%)	
Study selection in duplicate			0.541
No	8 (29.6%)	18 (23.7%)	
Yes and partial yes	19 (70.4%)	58 (76.3%)	
Data extraction in duplicate			0.297
No	10 (37.0%)	37 (48.7%)	
Yes and partial yes	17 (63.0%)	39 (51.3%)	
Description of RoB assessment			0.322
No	-	5 (6.6%)	
Yes	27 (100%)	41 (93.4%)	
RoB assessment in duplicate			0.696
No	10 (37.0%)	25 (32.9%)	
Yes	17 (63.0%)	51 (67.1%)	
**Domain: Critical appraisal**			
RoB results within studies			1.000
No	3 (11.1%)	11 (14.5%)	
Yes	24 (88.9%)	65 (85.5%)	
Description of protocol deviations			0.170
No and unclear	19 (70.4%)	42 (55.3%)	
Yes and does not apply	8 (29.6%)	34 (44.7%)	
Presence of spin bias			0.165

No	19 (70.4%)	63 (82.9%)	
Yes	8 (29.6%)	13 (17.1%)	
Discussion addressing RoB			0.755
No	19 (70.4%)	51 (67.1%)	
Yes	8 (29.6%)	25 (32.9%)	
Limitations thoroughly addressed			0.651
No	2 (7.4%)	4 (5.3%)	
Yes (both for study and review levels, only for study/outcome, only for review)	25 (92.6%)	72 (94.7%)	

ab: abstract; PICOS: acronym for Population, Intervention, Comparator/Control, Outcome, Setting; RoB : risk of bias.

*$p < 0.05$
